# Pesticides in a case study on no-tillage farming systems and surrounding forest patches in Brazil

**DOI:** 10.1038/s41598-021-88779-3

**Published:** 2021-05-10

**Authors:** Karlo Alves da Silva, Vitoria Beltrame Nicola, Rafaela Tavares Dudas, Wilian Carlo Demetrio, Lilianne dos Santos Maia, Luis Cunha, Marie Luise Carolina Bartz, George Gardner Brown, Amarildo Pasini, Peter Kille, Nuno G. C. Ferreira, Cíntia Mara Ribas de Oliveira

**Affiliations:** 1 Programa de Pós-Graduação em Gestão Ambiental, Universidade Positivo, Curitiba, 81280-330 Brasil; 2 Graduação em Biomedicina, Universidade Positivo, Curitiba, 81280-330 Brasil; 3 Programa de Pós-Graduação em Ciências do Solo, Universidade Federal do Paraná, Curitiba, 80035-050 Brasil; 4 Department of Life Sciences, Centre for Functional Ecology, University of Coimbra, Calçada Martim de Freitas, 3000-456 Coimbra, Portugal; 5 School of Applied Sciences, University of South Wales, Pontypridd, CF37 4BD Wales UK; 6 Embrapa Florestas, Colombo, Paraná 83411-000 Brasil; 7 Departamento de Agronomia, Universidade Estadual de Londrina, Londrina, 86057-970 Brasil; 8 School of Biosciences, Cardiff University, Cardiff, CF10 3AX Wales, UK

**Keywords:** Environmental sciences, Risk factors

## Abstract

With the growing global concern on pesticide management, the relationship between its environmental recalcitrance, food security and human health has never been more relevant. Pesticides residues are known to cause significant environmental contamination. Here, we present a case study on long-term no-tillage farming systems in Brazil, where Glyphosate (GLY) has been applied for more than 35 years. GLY and its main breakdown product, aminomethylphosphonic acid (AMPA) were determined in topsoil (0–10 cm) samples from no-tillage fields and nearby subtropical secondary forests by high-performance liquid chromatography coupled with a fluorescence detector. In addition, the presence of carbamates, organochlorines, organophosphates and triazines were also screened for. GLY and AMPA were present in all soil samples, reaching values higher than those described for soils so far in the literature. A significant decrease for AMPA was observed only between the secondary forest and the farm's middle slope for site B. GLY and AMPA were observed respectively at peak concentrations of 66.38 and 26.03 mg/kg soil. GLY was strongly associated with forest soil properties, while AMPA associated more with no-tillage soil properties. Soil texture was a significant factor contributing to discrimination of the results as clay and sand contents affect GLY and AMPA retention in soils. This was the first study to report DDT and metabolites in consolidated no-tillage soils in Brazil (a pesticide fully banned since 2009). Based on human risk assessment conducted herein and the potential risk of GLY to local soil communities, this study offers a baseline for future studies on potential adverse effects on soil biota, and mechanistic studies.

## Introduction

Globally, an average of 4 × 10^6^ tons of pesticides per year is used in agriculture, corresponding approximately to 0.27 kg pesticide/ha applied per year over the entire land surface^1^. Due to adverse effects on non-target organisms, pesticide application (linked to the expansion of agriculture) is one of the leading causes for natural habitat loss and, consequently, extinction of key functional species for ecosystem services^2^. Furthermore, although pesticide use can increase agricultural production, its potential impacts on environment quality, food safety and human health have raised serious concerns^3,4^.

Brazil is one of the largest food producers globally, and at the same time, one of the highest pesticide consumers. Between 2000 and 2014^5,6^, pesticide use in Brazil increased by 135%, and the most recent numbers show an increase up to 4 × 10^5^ tons per year^7^. Glyphosate (GLY) is the most widely commercialised herbicide accounting for 25% and 52% of the total pesticide application worldwide and in Brazil (in terms of volume), respectively^6,8^.

The interaction between soils and pesticides is driven by their inherent physical and chemical properties^9^. For instance, the adsorption of contaminants in the colloidal fractions and soil pore water availability are influenced by pH, mineral composition, organic matter content, and cation exchange capacity^10,11^. Due to their high toxicity and persistence in the environment^12^, organochlorines such as dichlorodiphenyltrichloroethane (DDT) and hexachlorobenzene (BHC) are prohibited in more than 70 countries^13^. Volatile organic pesticides, such as DDT, are transformed from the liquid/solid to the vapour state, increasing their dispersion, condensation, and precipitation in a soil-atmosphere exchange system^14–16^. Semi-volatile pesticide residues have been reported from agricultural soils worldwide (e.g., Malaysia^17^, India^18,19^, China^20–22^, Kenya^23^, European Union^24^ and Mexico^25^), but also in forest soils (e.g. Tibet^26,27^, China^28^, Uganda^29^, Republic Czech^30^, England^31^, Argentina^32^ and Brazil^33^).

Contaminants with high vapour pressure and low octanol–water partition coefficients, such as GLY and its primary metabolite, aminomethylphosphonic acid (AMPA) are described as persistent in certain types of soils^34^ and tend to remain strongly adsorbed at binding sites^10,35,36^. The AMPA half-life is much longer (up to 958 days) than GLY (up to 280 days) and depends on environmental conditions, which may speed or slow decomposition^37,38^. They can occur in the environment due to spray drift^39–42^, water and wind erosion of fine fractions of soil aggregates^36,43,44^, and precipitation of particulate matter^45–47^. GLY and AMPA have been reported in soils worldwide (e.g., Europe, USA, China, Egypt, Argentina)^24,48–51^, mostly in agricultural systems. Maximum concentrations of GLY reported range from 0.42 (Egypt) to 8.1 mg/kg soil (Argentina), while those of AMPA range from 0.34 (USA) to 38.9 mg/kg soil (Argentina) in cropping systems. In forest soils, they have been found in *Eucalyptus* plantations in Spain (maximum of 6.9 mg GLY/kg in soil and 0.77 mg AMPA/L in soil liquid phase)^52^ and by Newton et al.^48^ in several North America native forests (maximum of 4.6 mg GLY/kg, and 0.51 mg AMPA/kg soil). However, in native tropical and subtropical forest soils, particularly near cropping systems, there is still a lack of information for GLY and AMPA residues.

GLY is a multi-purpose desiccant herbicide widely used in annual and perennial crops, cover crops, horticulture and forestry, as well as in non-agricultural purposes (e.g., margins of highways and railways, sidewalks, parks)^53^. With the development of GLY-resistant genetically modified crops in the last 20 years, GLY use has become an integral part of weed control in over 100 million hectares worldwide^54^ and accounts for about 56% of global GLY use^8^. Most soybean and maise crops grown worldwide are now GLY-resistant varieties^55^. However, inappropriate agricultural practices and overuse of this herbicide have led to the emergence of GLY-resistant weeds^55^. Conversely, the widespread use of glyphosate was also helpful in the worldwide expansion of no-tillage agriculture, particularly in Brazil, where this soil-conservation practice occupies over 32 million hectares, almost 60% of the annual crop area of the country^56^.

This study performed a pesticide screening in no-tillage farming systems (NT) where pesticides have been regularly applied (> 35 years) and in surrounding fragments of secondary Atlantic forest (SF) in intermediate to advanced regeneration state. The study also evaluated the relationships between GLY and AMPA concentrations and soil’s physical and chemical characteristics and performed a human health risk assessment using an indirect probabilistic risk model. We hypothesised that location along the catena influences the concentrations of pesticide residues (e.g. by runoff or wind dispersion) with higher residue levels at sites located at the bottom of the slope when compared to the top.

## Results

### Soil physical and chemical properties

**Figure 1:**
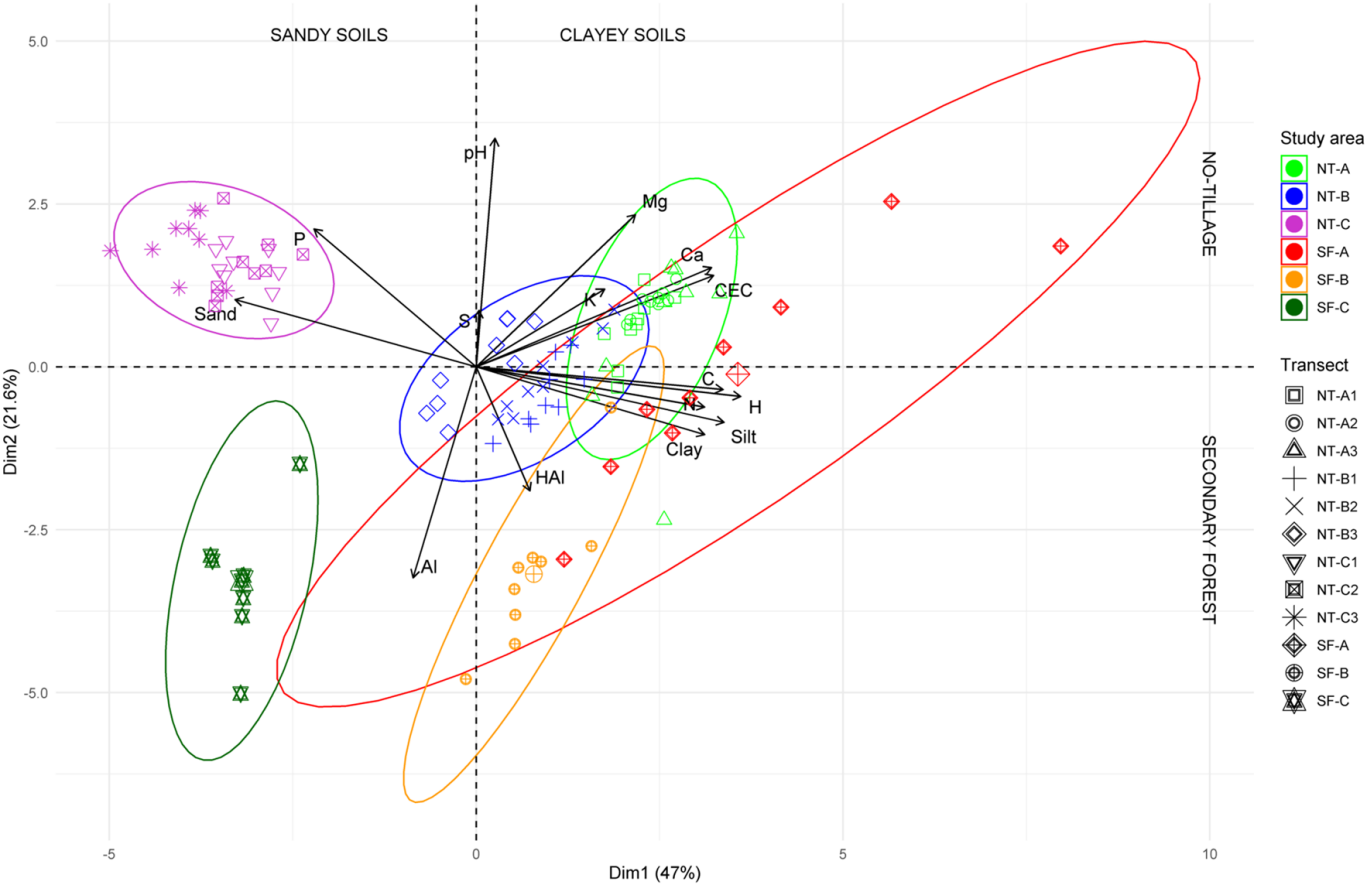
No-tillage farm (NT) and secondary forests (SF) soil properties. Principal component analysis (PCA) for physical (sand, silt and clay content) and chemical (pH, C, H, N, S, Al^3+^, H^+^Al, Ca^2+^, Mg^2+^, K^+^, P and CEC values) properties of the three sampling areas (A, B e C) of NT and SF.

The physical and chemical soil properties are represented in Table 1, and details of sampling transects in Supplementary Table S1 (Supplementary data—SD). The PCA analyses showed a general Kaiser–Meyer–Olkin (KMO) value of 0.60, indicating that the sampling effort was acceptable (low collinearity among variables themselves). The positive scores of the first principal component (Dim1) corresponded to clayey soils and negative scores to sandy loam soils, this axis is related to soil texture (eigenvalue = 7.05; variability = 47.01%; Fig. 1). The second principal component (Dim2) showed positive scores related to high pH and P values found in NT, and negative scores with low pH, higher Al^3+^ and H^+^ Al values, mainly found in SF soils. This axis is related to land use/vegetation cover (eigenvalue = 3.25; variability = 21.64%). The scores of Dim1 (soil texture) showed significant differences among samples (KW = 94.47), showing higher values for SF-A soils among forests (*p* = 0.004) and NT-A soils among farms (*p* = 0.0001). The scores of Dim2 also showed significant differences among areas (KW = 78.80), with higher values for SF-A soils from forests (*p* = 0.004) and NT-C soils for farms (*p* = 0.0001).

**Table 1:** Soil physical–chemical properties and soil types of the three sampling areas (A, B and C) of no-tillage farms (NT) and secondary forests (SF). Data presents average values ± standard deviation and [minimum–maximum] values.

Parameter	Site A		Site B		Site C	
	NT	SF	NT	SF	NT	SF
N (%)	0.38 ± 0.05 [0.29–0.49]	0.66 ± 0.27 [0.51–1.36]	0.30 ± 0.03 [0.22–0.35]	0.43 ± 0.06 [0.35–0.54]	0.15 ± 0.05 [0.07–0.26]	0.25 ± 0.02 [0.22–0.29]
C (%)	5.43 ± 0.92 [4.05–7.33]	6.27 ± 2.77 [4.8–13.52]	3.37 ± 0.46 [2.24–4.09]	4.80 ± 0.92 [3.55–6.65]	1.52 ± 0.58 [0.48–2.66]	2.46 ± 0.20 [2.09–2.84]
H (%)	2.07 ± 0.07 [1.88–2.17]	2.07 ± 0.37 [1.79–3.03]	1.68 ± 0.29 [1.21–2.07]	1.85 ± 0.14 [1.64–2.09]	0.20 ± 0.16 [0.02–0.52]	0.29 ± 0.04 [0.22–0.38]
S (%)	0.27 ± 0.50 [0.06–2.07]	0.10 ± 0.01 [0.08–0.12]	0.05 ± 0.01 [0.04–0.07]	0.07 ± 0.03 [0.05–0.14]	0.22 ± 0.53 [0.04–2.30]	0.03 ± 0.00 [0.02–0.03]
pH CaCl_2_	5.34 ± 0.16 [4.83–5. 59]	5.20 ± 0.44 [4.43–5.87]	4.86 ± 0.19 [4.53–5.20]	4.12 ± 0.20 [3.90–4.59]	5.56 ± 0.24 [5.13–6.02]	3.85 ± 0.19 [3.46–4.15]
Al^3+^ (cmolc/dm^3^ soil)	0.06 ± 0.04 [0.03–0.20]	0.15 ± 0.20 [0.00–0.66]	0.14 ± 0.09 [0.0–0.38]	1.15 ± 0.61 [0.15–2.06]	0.03 ± 0.05 [0.00–0.18]	1.65 ± 0.66 [0.46–2.85]
H^+^Al (cmolc/dm^3^ soil)	6.74 ± 7.61 [3.70–44.60]	5.33 ± 1.82 [3.20–9.00]	5.59 ± 0.79 [4.30–7.20]	8.91 ± 1.39 [6.20–10.50]	3.14 ± 0.79 [2.00–5.40]	7.49 ± 1.30 [6.20–10.51]
Ca^2+^ (cmolc/dm^3^ soil)	7.72 ± 1.13 [5.00–9.83]	6.76 ± 4.37 [0.95––14.03]	4.27 ± 1.14 [2.58–7.28]	1.96 ± 1.22 [0.43–4.70]	1.67 ± 0.68 [0.25–2.73]	0.50 ± 0.55 [0.08–1.83]
Mg^2+^ (cmolc/dm^3^ soil)	2.44 ± 0.72 [0.32–3.68]	3.30 ± 1.27 [1.40–5.32]	1.96 ± 0.37 [1.40–2.60]	1.39 ± 0.49 [0.68–2.40]	1.99 ± 0.46 [0.64–2.80]	0.61 ± 0.35 [0.28–1.48]
K^+^ (mg/dm^3^ soil)	0.06 ± 0.01 [0.03–0.09]	0.02 ± 0.01 [0.01–0.03]	0.06 ± 0.01 [0.03–0.09]	0.03 ± 0.01 [0.02–0.05]	0.03 ± 0.01 [0.01–0.05]	0.01 ± 0.00 [0.01–0.02]
P (mg/dm^3^ soil)	19.46 ± 6.53 [8.70–35.20]	4.51 ± 1.83 [3.10–9.10]	33.76 ± 9.09 [16.40–58.30]	8.26 ± 6.65 [3.50–24.70]	59.40 ± 26.01 [8.20–109.40]	10.91 ± 1.55 [9.10–14.20]
CEC (cmolc/dm^3^ soil)	10.28 ± 1.54 [7.42–13.56]	10.23 ± 5.71 [3.03–19.16]	6.43 ± 1.37 [4.40–9.62]	4.53 ± 1.18 [3.18–7.30]	3.71 ± 0.90 [1.95–5.54]	2.76 ± 0.67 [1.82–3.78]
Clay (g/kg soil)	548.1 ± 61.7 [437.5–637.5]	713.9 ± 39.3 [662.5–762.5]	675.5 ± 65.2 [575.0–762.5]	673.6 ± 11.6 [662.5–687.5]	153.2 ± 54.3 [87.5–262.5]	178.6 ± 21.4 [150.0–212.5]
Silt (g/kg soil)	195.8 ± 19.0 [175.0–250.0]	230.6 ± 33.7 [162.5–275.0]	145.8 ± 28.0 [87.5–187.5]	186.1 ± 18.2 [162.5–212.5]	44.0 ± 28.5 [12.5–100.0]	91.1 ± 14.9 [62.5–112.5]
Sand (g/kg soil)	256.0 ± 56.8 [175.0–362.5]	55.6 ± 44.4 [12.5–150.0]	178.7 ± 82.8 [87.5–337.5]	140.3 ± 23.2 [100.0–175.0]	802.8 ± 78.6 [650.0–887.5]	730.4 ± 31.8 [687.5–787.5]
Texture	Clay	Clay	Clay	Clay	Sandy loam	Sandy loam

### Glyphosate (GLY) and aminomethylphosphonic acid (AMPA) residues

The highest GLY concentration in this study was found in a forest soil sample (SF-A), representing also the highest GLY concentration found in any site studied worldwide until the moment (66.38 mg GLY/kg soil—Table 2). The highest AMPA concentration detected was 26.03 mg/kg soil (SF-A).

**Table 2:** Glyphosate (GLY) and aminomethylphosphonic acid (AMPA) concentrations, AMPA:GLY ratio and total extracted glyphosate values (TEG) in no-tillage farm (NT) and secondary Atlantic forest soils (SF) sampled in sites A, B and C. Data presents average ± standard deviation and [minimum–maximum] values.

Site	mg GLY/kg soil	mg AMPA/kg soil	AMPA:GLY ratio	TEG
**A**				
NT-A1 (upper land, n = 9)	**10.4 ± 6.3**^**A**^ [0.5–19.9]	**5.7 ± 2.2**^**A**^ [< LOQ–7.6]	**0.7 ± 0.6**^**A**^ [< LOQ–2.2]	**19.1 ± 8.9**^**A**^ [0.6–30.9]
NT-A2 (mid slope, n = 9)	**15.4 ± 5.7**^**A**^ [7.6–21.6]	**8.0 ± 2.7**^**A**^ [3.5–12.5]	**0.5 ± 0.2**^**A**^ [0.4–1.1]	**27.6 ± 9.1**^**A**^ [13.0–40.7]
NT-A3 (lower slope, n = 9)	**9.9 ± 7.6**^**A**^ [< LOQ–27.9]	**5.9 ± 2.7**^**A**^ [< LOQ–9.7]	**0.7 ± 0.3**^**A**^ [0.3–1.2]	**18.9 ± 11.0**^**A**^ [< LOQ–42.7]
NT (n = 27)	**11.9 ± 6.8**^**#**^ [< LOQ–27.9]	**6.5 ± 2.7** [< LOQ–12.5]	**0.6 ± 0.4** [< LOQ–2.2]	**21.9 ± 10.2** [< LOQ–42.7]
SF (n = 9)	**16.1 ± 9.3**^**A**^ [0.2–66.4]	**6.3 ± 4.0**^**A**^ [1.0–26.0]	**0.9 ± 1.4**^**A**^ [0.1–4.5]	**25.6 ± 13.7**^**A**^ [1.7–42.5]
**B**				
NT-B1 (upper land, n = 9)	**16.4 ± 7.8**^**B**^ [0.5–24.0]	**8.4 ± 3.6**^**BC**^ [1.3–13.0]	**0.8 ± 0.8**^**B**^ [0.4–2.8]	**29.1 ± 12.6**^**B**^ [2.5–42.9]
NT-B2 (mid slope, n = 9)	**20.8 ± 4.0**^**B**^ [14.4–39.1]	**10.1 ± 2.8**^**C**^ [5.8–14.6]	**0.5 ± 0.1**^**BC**^ [0.4–0.7]	**36.1 ± 7.6**^**B**^ [23.3–44.7]
NT-B3 (lower slope, n = 9)	**14.4 ± 5.4**^**B**^ [8.4–24.3]	**7.1 ± 3.2**^**BC**^ [3.7–13.1]	**0.5 ± 0.1**^**BC**^ [0.4–0.6]	**25.1 ± 10.2**^**B**^ [14.1–41.7]
NT (n = 27)	**17.2 ± 6.3** [0.5–27.2]	**8.5 ± 3.4** [1.3–14.6]	**0.6 ± 0.5** [0.4–2.8]	**30.1 ± 11.0** [2.5–44.7]
SF (n = 9)	**18.1 ± 4.0**^**B**^ [3.0–23.7]	**5.4 ± 2.0**^**B**^ [1.1–8.1]	**0.3 ± 0.2**^**C**^ [0.1–0.6]	**26.3 ± 5.0**^**B**^ [19.7–36.0]
**C**				
NT-C1 (upper land, n = 9)	**12.7 ± 7.9**^**C**^ [0.8–28.1]	**6.6 ± 4.2**^**D**^ [< LOQ–13.9]	**0.5 ± 0.3**^**D**^ [< LOQ–1.1]	**22.6 ± 13.6**^**C**^ [0.9–49.3]
NT-C2 (mid slope, n = 9)	**11.3 ± 7.2**^**C**^ [0.6–20.7]	**4.9 ± 2.8**^**D**^ [< LOQ–9.6]	**0.4 ± 0.2**^**D**^ [< LOQ–0.9]	**18.7 ± 11.1**^**C**^ [0.7–32.8]
NT-C3 (lower slope, n = 9)	**12.6 ± 5.**^**C**^ [3.7–19.4]	**5.6 ± 1.7**^**D**^ [3.6–9.1]	**0.5 ± 0.3**^**D**^ [0.3–1.2]	**21.1 ± 7.4**^**C**^ [10.2–33.3]
NT (n = 27)	**12.2 ± 6.6** [0.6–28.1]	**5.7 ± 3.0** [< LOQ–13.90]	**0.5 ± 0.3** [< LOQ–1.2]	**20.8 ± 10.7**0.7–49.3
SF (n = 9)	**18.1 ± 10.3**^**C**^ [0.1–34.5]	**4.7 ± 2.2**^**D**^ [2.6–9.3]	**5.1 ± 14.4**^**D**^ [0.1–43.5]	**25.3 ± 11.2**^**C**^ [8.1–44.2]

Letters represent significant differences among NT-*1, NT-*2, NT-*3 and SF-*.

Significant difference were found between the different catena transects (NT-A, NT-B and NT-C, α = 0.05).

No significant differences were obtained among the three SF areas (SF-A, SF-B and SF-C).

There were no significant differences observed between the SF areas for GLY, AMPA, AMPA:GLY ratio or TEG (Table 2). For GLY concentrations, no significant differences were observed between transects and forests from the same area. Still, the comparison between the average of the combined transects showed area NT-B to be significantly different from NT-A and NT-C (F_2,79_ = 4.59; *p* = 0.02 and *p* = 0.03, respectively). For AMPA concentrations, NT-B2 was significantly different from SF-B (F_3,33_ = 5.53; *p* = 0.01), which also gave rise to a significantly higher average for the combined transects in area NT-B when compared to areas NT-A and NT-C (F_2,79_ = 5.43; *p* = 0.04 and *p* = 0.02, respectively). However, these differences were not reflected in the AMPA:GLY ratio. Nonetheless, significant differences were observed between NT-B1 and SF-B (F_3,33_ = 2.71; *p* = 0.003), but not among the combined transect values (NT—F_2,79_ = 4.42; *p* = 0.10). The TEG values showed significant differences among the combined transects among areas (NT), with NT-B being significantly higher than NT-A (F_2,79_ = 6.20; *p* = 0.01) and NT-C (F_2,79_ = 2.56; *p* = 0.02). The two-way ANOVA analysis showed no significant interactions between the transects and the land-use for both GLY (F_2,79_ = 15.59; *p* = 0.20) and AMPA residues (F_2,81_ = 11.14; *p* = 0.36).

### Screening pesticides occurrence

Due to technical difficulties, the initial pesticide screening of 54 parental compounds and metabolites could only be rerun after 480 days of storage. The long-term storage led to the degradation of compounds present in soils, and only the most persistent ones were detected. Since the concentration values would not reflect accurate concentrations found in the environment, only the presence/absence of these metabolites is provided in Table S2 (SD). Please note that the absence of a particular metabolite does not necessarily mean that it was not present but that its concentration was below detection limits. Traces of p,p′-DDT and its metabolite p,p′-DDE were found above the limit of detection in all NT areas. The metabolite p,p′-DDD was also present in NT-A.

### Relationships between soil properties, GLY and AMPA

**Figure 2:**
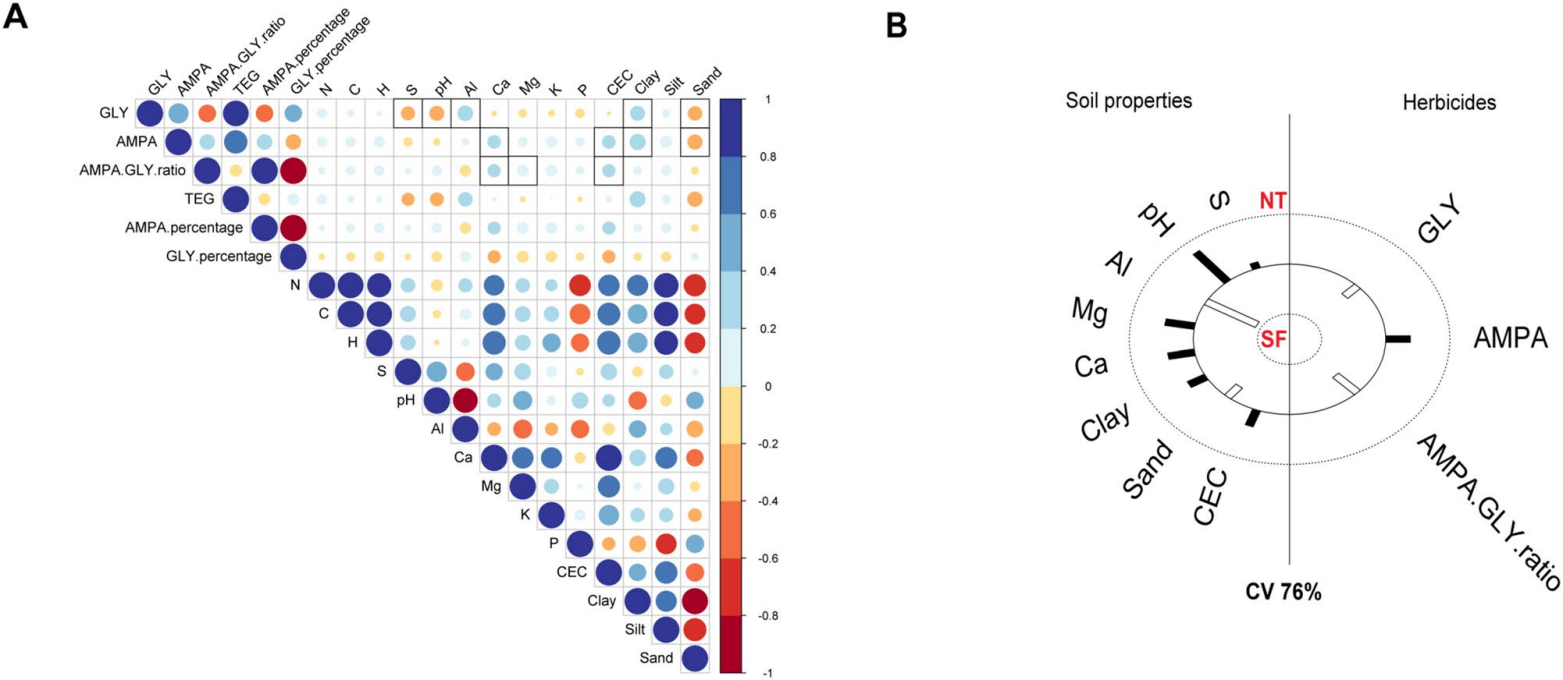
Linear regression between pesticide values and soil properties. (**A**) Correlogram showing the relationship among physical–chemical soil properties and GLY and AMPA values. Positive correlations are displayed in blue and negative correlations in red color. Color intensity and the size of the circle are proportional to the correlation coefficients. (**B**) Canonical variation (%) of the principal axis on canonical correlation analysis among soil properties and pesticides values using the significant correlate parameters. *NT* no-tillage farms, *SF* secondary forest.

According to the linear regression (Fig. 2A), GLY concentrations were positively correlated with Al^3+^ and clay content and negatively correlated with S, pH and sand content. On the other hand, AMPA concentrations showed positive correlations with Ca^2+^, CEC and clay content and negative correlations with sand content. AMPA:GLY ratio showed a positive correlation with Ca^2+^, Mg^2+^ and CEC. TEG showed positive correlations with Al^3+^, clay content and negative correlations with S, pH and sand content (Table S3—SD). In the canonical correlation analysis (Fig. 2B), the principal axis represented 76% of the total canonical variation (F = 1.6, *p* = 0.04), with GLY and AMPA:GLY ratio correlating with SF soils while AMPA correlated with NT soils.

**Figure 3:**
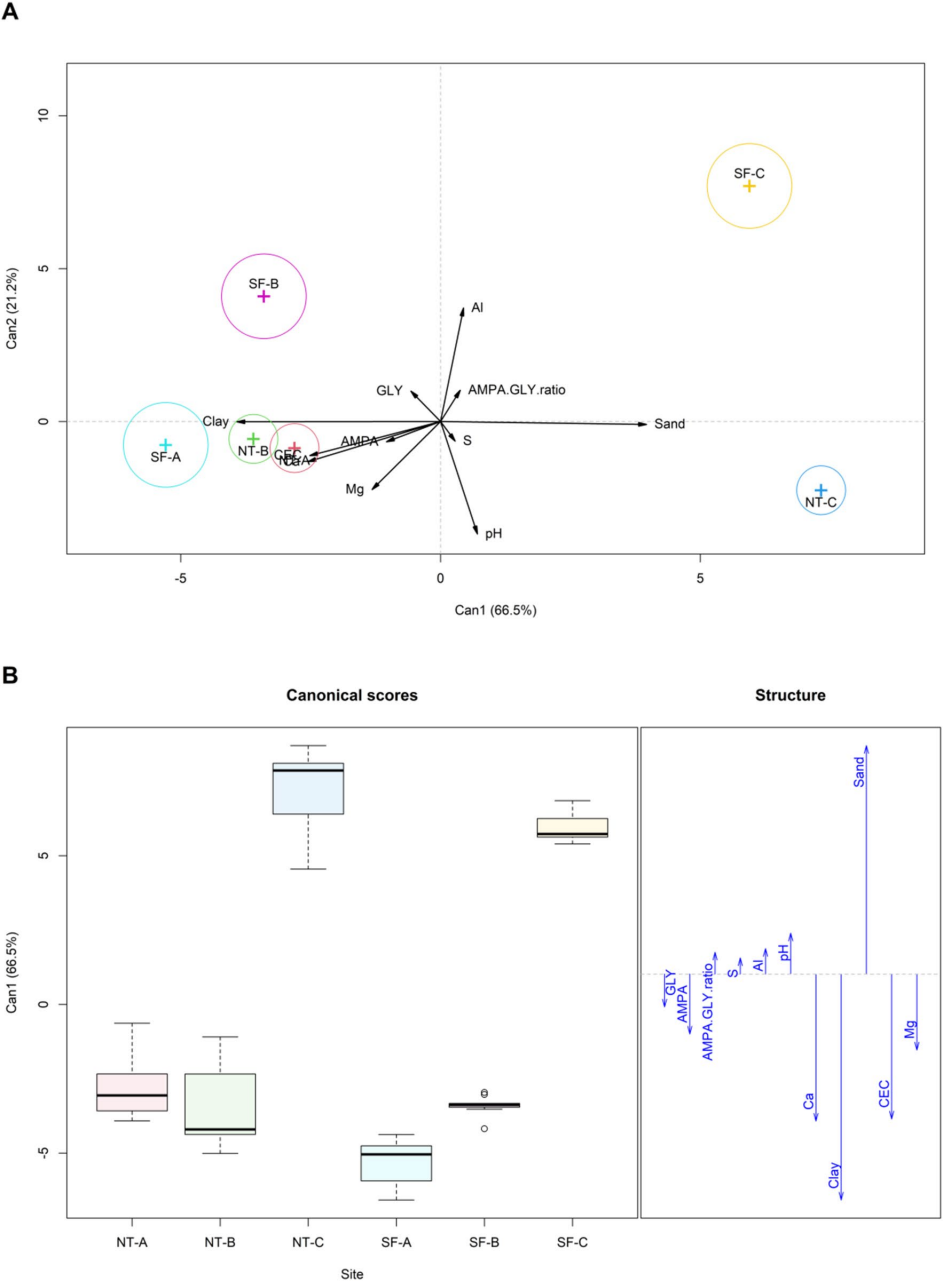
Maximum canonical discriminant function among study sites explaining 87.08% in two canonical axes. (**A**) Generalised canonical discriminant analysis to identify vector behaviour in relation to study sites in a multivariate linear model plotted in canonical space. (**B**) Unfolding of the principal axis (Can1) of canonical discriminant analysis from summarised modelling of canonical discriminant function, seeking linear combinations of quantitative variables to identify the factors that separate groups to the maximum. *A, B, C* study sites; *NT* no-tillage; *SF* secondary forest.

In the canonical discriminant analyses (Fig. 3A), the principal discriminant axis (Can1) explained 66.7% of the maximisation between groups. This axis seemed to discriminate the samples according to soil properties, mainly between sandy and clayey soils (F = 36.71, *p* < 0.0001; Fig. 3B). Additionally, it differentiated GLY and AMPA concentrations that were dependent on clay and sand content. This was confirmed by the TEG values that showed a positive correlation to clay and a negative correlation to sand contents. The axis Can2 explained 21.1% of the maximisation between groups, suggesting a separation between soil types, with NT-farming systems appearing in the positive component and SF-systems in the negative (with the exception of SF-A).

### Human risk assessment

The indirect human risk assessment was calculated based on the incremental lifetime cancer risk (ILCR) values presented in Table 3. The model that estimates the probabilistic risk from generic characteristics was built for adults between 18 and 70 years old. Even considering the uncertainties of the indirect exposure assessment model, the results showed that GLY concentrations in the areas sampled may represent a carcinogenic risk to public health since they are higher than the reference risk value of 1 × 10^–4^.

**Table 3:** Incremental lifetime cancer risk (ILCR) from average daily dose (ADD, mg/kg/day) by four exposure routes. Soil intake (ADDIngSoil), food intake (ADDIngFood), dermal contact (ADDDerm) and inhalation (ADDInhal). SF values represent the oral slope factor (SFo) and dermal contact (SFabs). IUR represents the inhalation unit risk (unitless).

Site	ADDIngSoil	ADDIngFood	ADDDerm	ADDInhal	SFo (soil)	SFo (vegetable)	SFabs	IUR	ILCR
**A**									
NT-A1	16 ± 10 [1–30]	41,452 ± 25,114 [2160–21.6]	28 ± 17 [1–53]	50 ± 30 [3–96]	0.01 ± 0.01 [0.00–0.02]	26 ± 16 [1–49]	0.02 ± 0.01 [0.00–0.03]	0	26 ± 16^**A**^ [1–48]
NT-A2	23 ± 9 [11–33]	61,425 ± 22,664 [30219–86021]	41 ± 17 [20–57]	74 ± 27 [36–104]	0.01 ± 0.01 [0.01–0.02]	38 ± 14 [19–53]	0.03 ± 0.01 [0.01–0.04]	0	38 ± 14^**A**^ [19–53]
NT-A3	15 ± 11 [0–42]	39,569 ± 30,068 [92–111269]	26 ± 20 [0–74]	48 ± 36 [0–134]	0.01 ± 0.01 [0.00–0.03]	25 ± 19 [0–69]	0.02 ± 0.01 [0.00–0.05]	0	25 ± 19^**A**^ [0–69]
SF	24 ± 14 [0–42]	64,073 ± 36,908 [860–111817]	43 ± 24 [1–74]	77 ± 45 [1–135]	0.02 ± 0.01 [0.00–0.03]	40 ± 23 [1–69]	0.03 ± 0.02 [0.00–0.05]	0	40 ± 23^**A**^ [1–69]
**B**									
NT-B1	25 ± 12 [1–36]	65,279 ± 30,994 [1901–95658]	43 ± 21 [1–63]	79 ± 37 [2–115]	0.02 ± 0.01 [0.00–0.02]	40 ± 19 [1–59]	00.3 ± 0.01 [0.00–0.04]	0	41 ± 19^**A**^ [1–59]
NT-B2	31 ± 6 [22–41]	82,652 ± 17,759 [57505–108463]	55 ± 10 [38–72]	100 ± 19 [69–131]	0.02 ± 0.00 [0.01–0.02]	51 ± 10 [36–67]	00.3 ± 0.01 [0.02–0.04]	0	51 ± 10^**A**^ [36–67]
NT-B3	22 ± 8 [13–37]	57,227 ± 21,571 [33448–96904]	38 ± 14 [22–64]	69 ± 26 [40–117]	0.01 ± 0.01 [0.01–0.02]	35 ± 13 [21–60]	00.2 ± 0.01 [0.01–0.04]	0	36 ± 13^**A**^ [21–60]
SF	27 ± 6 [16–36]	72,092 ± 15,820 [41800–94193]	48 ± 10 [28–63]	87 ± 19 [50–114]	0.02 ± 0.00 [0.00–0.03]	45 ± 10 [26–68]	00.3 ± 0.01 [0.02–0.04]	0	45 ± 10^**A**^ [26–58]
**C**									
NT-C1	19 ± 12 [1–42]	50,388 ± 31,291 [3214–111961]	33 ± 21 [2–74]	61 ± 38 [4–135]	0.01 ± 0.01 [0.00–0.03]	31 ± 19 [2–69]	00.2 ± 0.01 [0.00–0.05]	0	31 ± 19^**A**^ [2–69]
NT-C2	17 ± 11 [1–31]	44,990 ± 28,694 [2447–82342]	30 ± 19 [2–55]	54 ± 35 [3–99]	0.01 ± 0.01 [0.00–0.02]	28 ± 18 [2–51]	00.2 ± 0.01 [0.00–0.03]	0	28 ± 18^**A**^ [2–51]
NT-C3	19 ± 8 [6–29]	50,131 ± 20,693 [14675–77162]	33 ± 14 [10–51]	61 ± 25 [18–93]	0.01 ± 0.00 [0.00–0.02]	31 ± 13 [9–48]	00.2 ± 0.01 [0.01–0.03]	0	31 ± 13^**A**^ [9–48]
SF	27 ± 15 [0–52]	72,069 ± 40,966 [482–137537]	48 ± 27 [0–91]	87 ± 49 [1–166]	0.02 ± 0.01 [0.00–0.03]	45 ± 25 [0–85]	00.3 ± 0.02 [0.00–0.06]	0	45 ± 25^**A**^ [0–85]

Data presents the average ± standard deviation and [minimum–maximum] values. Different letters represent a significant difference.

## Discussion

For the first time, GLY and AMPA were quantified in no-tillage farming soils and surrounding subtropical secondary forest soils in Brazil. GLY and AMPA's ubiquitous occurrence is in agreement with other studies in South America (e.g. Aparício et al.^51^ and Primost et al.^34^). GLY concentrations observed in all sites were higher than those reported worldwide (e.g. Egypt: 0.42 mg/kg soil^50^, Portugal 1.14 mg/kg soil^36^, US: 4.67 mg/kg soil^57^ or Argentina: 2.30 mg/kg soil^34^). As for the AMPA concentrations in the present study, they are also higher than those found in Portugal (0.73 mg/kg soil)^36^, US (0.18 mg/kg soil)^58^ or Argentina (4.20 mg/kg soil)^34^, but lower than maximum values reported for the latter country^34^. Linear correlation and regression analyses confirm the pseudo-persistence hypothesis attributed to AMPA due to maximum soil half-life^34^, the continuous GLY application and the maximum half-life of the precursor molecule.

The hypothesis that soil in lower areas of the catena would show higher concentrations of pesticides (i.e., due to runoff), was rejected. No differences were found in either GLY or AMPA concentrations at upper, middle and lower transects. The hypothesis was based on the mobility of these compounds^36^ and studies in Argentina^59^ and China^49^ that showed the hypothesised pattern. The rejection of the hypothesis may be related to the sampling period (dry season), in a year with few rainy days and highly reduced rainfall.

Only at one site (B) significant differences in AMPA levels were detected between the forest (SF-B) and the middle slope (NT-B2). This finding may be related to higher pH, Ca^2+^ and P levels in NT. The higher levels of P in NT-B2 to SF-B may influence the mineralisation of pollutants in the soil and explain the high levels of AMPA in this transect. Previous studies using microcosms indicate that phosphate levels can cause negative regulation of carbon-phosphorus lyase (C–P lyase)^60,61^, an enzyme responsible for degrading GLY and AMPA. Additionally, the increase in soil fertility through the use of fertilisers with phosphate over a long time promotes P competition with the GLY phosphonate group for soil binding sites, as reported by Gimsing et al.^10^ and Munira et al.^62^.

Previous literature showed that pesticide degradation by microorganisms is lower in soils with more acid pH^58^. Nonetheless, our results show that it is not the most important factor explaining GLY and AMPA levels in NT and SF. The availability of GLY molecules increases as pH increases as a result of lime application^63^ and mineral fertilization^64^. However, these practices may become hazards to surrounding areas due to the sorption and desorption dynamics of modified glycine^63^. Liming in NT farms promotes an electrostatic repulsion of GLY due to excess negative electric charges in the soil, which reduces the formation of hydrogen bonds and releases GLY from the chelating reaction^63^. The result is reduced sorption and increased availability of the precursor molecule for degradation, which may explain AMPA concentrations in NT sites. The AMPA:GLY ratio showed a negative correlation to soils with high pH, thus supporting a higher degradation of GLY observed in NT farms. The AMPA:GLY ratio values in forest soils indicate aged mixtures and slow degradability of older GLY, according to Lupi et al.^63^ and Primost et al.^34^. To explain the observed slow degradability of GLY in forest soils, further studies are needed. For example, it would be beneficial to determine whether the high concentrations of AMPA found in soils under NT practices may be also associated with the diversity and role of microbial communities present (e.g., *Pseudomonas* spp. and *Agrobacterium* spp.). These communities may encode active ingredient enzymes (e.g. oxidoreductases)^65^ that use GLY as a source of carbon, nitrogen and phosphorus^66^.

GLY sorption is favoured in acidic environments^63,67^, due to the protonated soil solution that promotes the formation of different GLY speciation forms with a positive net charge, facilitating the formation of GLY functional group complexes (R-NH, R-COOH and R-PO(OH)) with the metals present in those soils^68,69^. Previous studies^10,70–72^ consider that amorphous metal oxides and hydroxides play a major role in GLY sorption. The solubility and availability of Al^3+^ of the cation exchange complex are pH-dependent, and the levels of Al^3+^ and H^+^ Al observed in the SFs of the present study showed a negative correlation with pH. This raises the hypothesis that GLY retention in forest soils increases the half-life of this active product, reducing availability for biological degradation. This can be the result of the forest environmental characteristics, with high metal ion contents, acidic pH, and reduced light and higher C sources. The mechanism of GLY retention in forests may represent a slow and continuous source of AMPA for environmental compartments, highlighting, therefore, the need to preserve the stability of forest systems due to their buffering effects on environmental contaminants. As presented before, GLY and AMPA mobility in the environment can be explained by spray drift, water or wind erosion. Their detection in high concentrations in SF may result from herbicide applications in all neighbouring agricultural areas. Future studies may help better explain this transport and deposition in non-target areas, without intentional application.

In the present study, soil texture was the factor with the highest discriminant variation. According to the literature, GLY mobility is influenced by soil texture^11,63^, with clayey soils being less favourable for mobility^73^, while sandy soils increase transport phenomena^74^ and vertical mobility^52^ to groundwater^75^. In addition to the reduced content of metal oxides (for complexation of the R-PO(OH) group with soil colloids), the speed of water flow in macropores of sandy soils influences the transport of bound and unbound contaminants. Fast flow reduces the time for adsorption (non-instantaneous process) of GLY with the soil matrix in equilibrium^74^. The present case study represents a first but important result concerning the study and detection of pesticide residues in NT and neighbouring native forests. Future studies are needed in order to verify whether these phenomena also occur in different soil types and in other Brazilian biomes. This soil contamination poses a risk not only to food quality and human health (since these compounds have been identified as endocrine disruptors) and ecological processes for ecosystem services maintenance^76–79^. GLY and AMPA presence in soil may promote toxicity to key species for biodiversity conservation^80^, which are fundamental for maintaining the interactions that ensure environmental services.

In addition to the risks to soil organisms, GLY is also considered a probable carcinogen for humans^81^. In this sense, the use of tools that can assess risk of humans’ exposure is of utmost importance. These tools can be used directly, indirectly and from reconstructed scenarios. According to EFSA^82^, chronic exposure of pure GLY concentrations at the rate of 1.4 mg GLY/kg/day increased the incidence of malignant lymphomas in rats, while chronic exposure of parental rabbits (1 mg GLY/kg/day) influenced offspring development due to delayed ossification and increased skeletal and cardiac malformations. In a recent review, Van Bruggen et al.^83^ pointed out to the positive correlations between GLY use and increased diseases such as attention deficit hyperactivity disorder (ADHD), kidney disease, Alzheimer's and Parkinson's, abortions, and dermatological diseases. In previous studies, continuous low exposure (70 mg GLY/kg/day) was suggested to promote neurotoxic effects by altering acetylcholinesterase activity^84^. GLY increased production of reactive oxygen species (> 42 mg GLY/L), leukocyte DNA damage (85–1690 mg GLY/L), and reduced DNA methylation (42 mg GLY/L) have been reported in vitro^83^. Even if the concentrations that presented risk in vitro are higher than those obtained in the present study, the contamination levels observed in the areas can be considered a problem of global concern. When calculating the human risk equations, oral intake was one of the most important factors contributing to the risk. Its importance is even greater if consider that the limits of GLY in food such as coffee (1 mg/kg BR vs 0.1 mg/kg EU), sugar cane (1 mg/kg BR vs 0.05 mg/kg EU), soybean (10 mg/kg BR vs 0.05 mg/kg EU) and drinking water (0.5 mg/L BR vs 0.0001 mg/L EU) permitted in Brazil are well above the limits accepted by the EU^6^. Despite the human health risk assessment having uncertainties and limitations due to generalisations^85^, the results obtained from the present model should be taken as a warning, and should be used to review GLY application regulations and policies. In countries such as Luxemburg, Vietnam, Sri Lanka and El Salvador, for example, GLY has already been banned, since it can trigger chronic kidney disease of unknown etiology in farmers^86^.

The occurrence of p,p′-DDT, p,p′-DDD and p,p′-DDE in soils at the sample sites also represents a risk to ecosystems, based on their lipophilic profiles (log Kow > 3). After a decade of banning DDT in Brazil (Law n. 11.936/2009), these results confirm the long-term persistence and dispersion of these pollutants in the environment, particularly considering there the lack of history of DDT application at all of the farms evaluated (at least over the last 35 years). According to the Food and Agricultural Organisation (FAO, 2000)^87^, depending on the microbiological activity and abiotic variables, DDT has a half-life up to 30 years in the soil, generating secondary metabolites (e.g. p,p′-DDD, p,p′-DDE) until total mineralisation and equilibrium in soil-atmosphere exchanges are reached^88^. Due to the recalcitrant potential and long half-life, these compounds have been classified as Persistent Organic Pollutants under the Stockholm Convention in 2001^89^, being distributed all over the planet^90–92^.

Despite the adoption of soil conservation practices like no-tillage that result in important environmental benefits, the use of more sustainable and less-intensive pest management techniques need to be further explored and developed for widespread use on-farm in order to reduce dependence on chemical pesticides. Current intensive agricultural practices require pesticide application in most cases, but the high residual levels found, highlight the need for further studies on the reasons for the high persistence of both GLY and AMPA in soils. Additionally studies on their toxicity to soil organisms, the use of less persistent alternative herbicides and even practices like improved weed management (e.g., mechanical weed control or rotation of different herbicide formulations) can provide important complementary information. Finally, our results also reflect the need to update the Brazilian Resolution 420 of the National Environmental Council (CONAMA/420)^93^, in order to review guideline values for contaminants such as DDT. As for GLY and AMPA residues in Brazilian soils, at the moment, there is no legislation which limits the maximum concentration in soils or number of applications. This is particularly concerning considering the frequency of applications each year (three times at the farms studied), and because every five spraying events was estimated to result in the increase of 1 mg GLY/kg soil in farms in neighbouring Argentina^34^.

## Conclusion

This study collected soil from three different farming areas and nearby forest patches to perform a pesticide screening. This is the first study in Brazil that quantified GLY and AMPA in soils under NT farming practices and surrounding subtropical SF. GLY and its primary metabolite AMPA were found in all studied areas, and GLY concentrations are the highest ever reported in the world. Based on the high carcinogenic indices calculated in the present study, such concentrations may promote adverse effects on human metabolic homeostasis and soil quality-related organisms. The present study indicates that land-use and soil type can influence the retention of such contaminants, since acidic soils with high metal contents increase GLY adsorption. Future studies should investigate the possible mechanisms and transport pathways of GLY and AMPA from the agricultural land to forest areas. Soil texture also discriminated the maximum variation between the study sites, highlighting the importance of clay and sand contents to GLY and AMPA availability. This study can provide evidence that supports limits for pesticide applications based on different soil types in Brazil. Environmental legislation, such as the CONAMA/420 resolution, requires urgent revision as the values and guiding criteria for pesticides residues in soils report only to commercially prohibited products but not for commercially approved pesticides (no guiding values exist for GLY and AMPA in soils). DDT and metabolite residues in NT soils confirmed the persistence of these compounds due to high environmental stability. Despite the impossibility to show actual values, this is the first study to report DDT and metabolites in consolidated no-tillage soils in Brazil. Faced with the potential risk to soil communities, future studies using ecotoxicological assays are needed to determine its bioavailability and to understand the mechanisms of toxicity to non-target organisms, essential for the maintenance of ecosystem services.

## Methods

### Study area

The study areas were selected due to the long-term history of pesticide use (> 35 years) under no-tillage farming system (NT) in the State of Parana, Brazil. Study area details are shown in Table 4, and rainfall conditions are showed in Table S4 (SD). The sampled areas (A, B and C) are located in the region with the largest number of pesticide-applied rural establishments (63%) and the second most sprayed per hectare per year in Brazil (9.81 mg pesticides/kg soil)^6^. The sampling sites' exact locations are not included due to privacy reasons but may be provided upon request to the authors and conditioned to acceptance by the owners. The coordinates provided correspond to the closest settlement. The herbicide GLY was applied on average three times a year, with 7.7 L/ha/year or 2.16 mg active ingredient/kg soil/year, and was at least sprayed one month before soil sampled in site NT-A and NT-B and the previous year in NT-C. In NT, GLY had a maximum application rate of three times per year.

**Table 4:** Description of the main characteristics of the three agroecosystems.

Site	Above sea level (m)	Distance between NT and SF (m)	Geographic positioning (closest settlement)	Start of NT	Soil classification (FAO)	Köppen climate	Field or forest size (ha)	Vegetation and crop rotation details	Commercial products applied^b^ (no.)	Annual doses applied for GLY (l/ha/year/mg pesticides/kg soil/year)
NT-A	759	850	23° 56′ 9.42" S 51° 20′ 13.50"W	1982	Ferralsol	CFa	27	C^a^: soybean, corn, white oats CC: brachiaria	H: 8 F: 4 I: 5 O: 2	8.2/1.97
SF-A	782			–	Ferralsol	CFa	42	Transition of ombrophilous mixed forest and seasonal semideciduous forest	–	–
NT-B	978	1877	23° 54′ 14.11" S 51° 13′ 24.15" W	1974	Ferralsol	CFa	23	C: soybean, corn, wheat, white oats CC: black oats	H: 6 F: 5 I: 7 O: 2	7.0/1.68
SF-B	1021			–	Ferralsol	CFa	5	Transition of ombrophilous mixed forest and seasonal semideciduous forest	–	–
NT-C	878	1250	25° 25′ 34.87" S 50° 0′ 12.39" W	1976	Cambisol	CFb	42	C: soybean, corn, wheat, white oats CC: black oats	H: 20 F: 17 I: 18 O: 3	8.0/2.56
SF-C	888			–	Cambisol	CFb	9	Ombrophilous mixed forest	–	–

*NT* no-tillage farming system, *SF* secondary forest fragment, Soil classification, according to FAO^94^ and climate classification, according to Köppen-Geiger (1936): *CFa* humid subtropical climate, *CFb* temperate oceanic climate. All these areas are found within the Atlantic forest biome.

^a^Crops (C) and cover crops (CC) planted over the last 4 years.

^b^Number of commercial products applied at the site over the last 4 years; *H* Herbicides, *F* Fungicides, *I* Insecticides, *O* others (for seed treatment and growth regulators).

Soil sampling was carried out in June 2018, in three transects approx. 150–200 m distant from each other in an altitudinal gradient (1—upper area; 2—middle slope; 3—lower area) along the catena at each NT farm, and in one transect in a nearby secondary forest (SF; Figs. S3, S4 and S5 in SD). The secondary forest resulted from a selective logging of the native forests have been under a process of natural regeneration for more than 50 years (information provided by the farmers). A total of nine samples distant 20 m apart were collected in each transect. To avoid a possible edge effect, the first soil sampling point in each SF transect was taken at least 20 m from the forest margin (minimum distance at site B). The maximum distance of a particular forest sample from a neighbouring agricultural field was approx. 130 m (site A). Soil samples were collected according to the Tropical Soil Biology and Fertility method (TSBF)^95^ adapted by Bartz et al.^96^. Briefly, 25 × 25 cm monoliths were taken from the upper 10 cm soil layer. After removing the plant and litter (residue) cover from the soil to avoid any misleading quantification of pesticides adsorbed to organic matter, monoliths were individually bagged and transported in refrigerated boxes to the laboratory where they were stored at − 20 °C until pesticide screening.

### Soil quality assessment and pesticide screening

Soil chemical (pHCaCl_2_, Al^3+^, H^+^Al, Ca^2+^, Mg^2+^, K^+^, P, cation exchange capacity—CEC) and physical attributes (sand, silt, clay contents) were performed according to Marques and Motta^97^. Total C, H, N and S contents of the soil samples were determined using a CHNS Elemental Analyzer (Elementar Vario Macro Cube analyser), by combustion at 1150 °C, using He as the carrier gas. High-performance liquid chromatography quantified GLY and AMPA with a detection limit of 0.04 mg/kg soil for both analytes at the Universidade Estadual do Oeste do Parana in Cascavel, Parana. For this purpose, both GLY and AMPA analytes were determined using the 9-fluorenylmethyl chloroformate (FMOC-Cl) derivatisation method, described by Sun et al.^98^, associated with the removal of residual FMOC-Cl, described by Le Fur et al.^99^. Calibration curves were prepared using GLY (97% purity, Sigma Aldrich) and AMPA (99% purity, Sigma Aldrich) standards. An uncontaminated matrix sample from 1876, confirmed by Remor et al.^100^, was also analysed in triplicate after fortification procedures with standard solutions. Other details of the analytical procedure are shown in Supplementary Table S5.

Trace residues analyses of other semi-volatile organic pesticides were performed for the same soil samples. Due to technical problems, the initial analyses could only be repeated after a 480 days storage period at − 20 °C. The reanalyses were performed by Centro de Biologia Experimental Oceanus LTDA. Screening for the presence of semi-volatile organic pesticides (thirteen-one organochlorines, thirteen organophosphorus, six carbamates, two triazine, and one pyrethroid compounds along with their metabolites) were extracted by a sonication method, according to US EPA method 3550C^101^, followed by gas chromatography device coupled with mass spectrometry, according to US EPA method 8270D^102^.

### Data analysis

The matrices of physical–chemical properties of soils were analysed by Kaiser–Meyer–Olkin (KMO) criteria (KMO > 0.5), which evaluates the degree of colinearity among variables^103^. The scores resulting from the principal component analysis (PCA) were evaluated for significance using the Kruskal–Wallis test, performing the post-hoc Dunn's test, since the data displayed non-normal distribution (Shapiro–Wilk test)^104^.

GLY and AMPA concentrations were expressed in mg/kg soil. Samples with values bellow detection limit (LOQ < 0.04 mg/kg/soil) were replaced by randomised numbers data analyses (n = 5/108). The outliers were dropped based on the Cook's distance (d > 0.1, n = 6/108)^105^. The GLY and AMPA values were analysed per transect (upper, middle and lower area) and compared among themselves and with the respective forest in each study area. GLY and AMPA concentrations were also evaluated among NT farms (NT-A, NT-B and NT-C), and among forests (SF-A, SF-B, SF-C). As GLY and AMPA concentrations displayed normal distribution (Shapiro–Wilk test) and homoscedasticity of variance (Bartlett test)^106^, they were submitted to an analysis of variance (ANOVA) test followed by Tukey's post-hoc test. To identify possible pesticide mobility in the catena, two-way ANOVA analysis was used to test for interactions between NT slope position at each NT site using GLY and AMPA values.

The percentage of AMPA in relation to the total modified glycines was measured according to Eq. (1) proposed by Battaglin et al.^57^:

1 $$\% {\text{ AMPA}} = {\text{C}}_{{{\text{AMPA}}}} \left( {{\text{mg AMPA/kg}}\;{\text{soil}}} \right)/{\text{C}}_{{{\text{glyphosate}}}} \left( {\text{mg GLY/kg soil}} \right) + {\text{C}}_{{{\text{AMPA}}}} \left( {\text{mg AMPA/kg soil}} \right)*{1}00$$

The AMPA:GLY ratio Eq. (2) was applied to estimate the age of the mixtures, and low values of this ratio indicate the recent entry of the precursor molecule, while high values indicate aged mixtures, according to Lupi et al.^63^ and Primost et al.^34^:

2 $${\text{AMPA:GLY ratio}} = {\text{C}}_{{{\text{AMPA}}}} \left( {\text{mg AMPA/kg soil}} \right)/{\text{C}}_{{{\text{glyphosate}}}} \left( {\text{mg GLY/kg soil}} \right)$$

The total extracted GLY (TEG) from the soil samples was also calculated considering the AMPA concentration, expressed in GLY mass equivalent basis, added to the GLY concentration, according to the Eq. (3) proposed by Coupe et al.^107^:

3 $${\text{TEG}} = {\text{C}}_{{{\text{glyphosate}}}} + {\text{C}}_{{{\text{AMPA}}}} \times {\text{MW}}_{{{\text{glyphosate}}}} /{\text{MW}}_{{{\text{AMPA}}}}$$

where C_glyphosate_ and C_AMPA_ represent GLY and AMPA concentrations, respectively, MW_glyphosate_ represents GLY molar weight (169 g/mol), and MW_AMPA_ represents AMPA molar weight (111 g/mol).

Soil properties without correlation with GLY, AMPA and AMPA:GLY values according to Spearman's test were not used in the canonical correlation analysis (CCA). The canonical variation represents the values of chemical and physical parameters related to the herbicide concentrations. The eigenvalues represent a correlation between each pair of canonical root axes. A canonical discriminant function was created to separate the groups by maximising the variation between them to the variation within each group. Multivariate analysis of variance (MANOVA) and Pillai test were used as a preliminary test of canonical discriminant function to identify whether variations in treatment levels have a higher influence on data variance than error.

In all statistical tests, the level of significance was 0.05. All data analyses and figures were performed using R software version 4.0.3 (R Core Team, 2020. R: A Language and Environment for Statistical Computing. R Foundation for Statistical Computing, Vienna, Austria. http://www.R-project.org/)^108^, and statistical computing packages are provided in Table S6 (SD). The semi-volatile organic pesticides were not included in the statistical analysis due to the samples' long-term storage (480 days at − 20 °C).

### Human risk assessment

A generic probabilistic risk model was developed to estimate the carcinogenic risk of GLY concentrations found in the present study. The calculation of incremental lifetime cancer risk (ILRC) was performed according to the methodology described by Exposure Factors Manual^85^ and Generic Exposure Routes Assumptions and Data Source Document^109^ and is fully described in the Supplementary data. Details of the parameters used to estimate the risk of exposure to human health are presented in Table S7 (SD). Physiological and behavioural data related to human lifestyle were used according to Qu et al.^110^.

## Supplementary Information

Supplementary Information.

## Data Availability

This manuscript can be also accessed through http://orca.cf.ac.uk/140400/.
