# Supplementary material for: Pesticides in a case study on no-tillage farming systems and surrounding forest patches in Brazil
Source: Sci Rep. 2021 May 10;11:9839. doi: 10.1038/s41598-021-88779-3 (PMC8110586; doi:10.1038/s41598-021-88779-3)
Supplement: Supplementary file 1 — Supplementary Information. [file 41598_2021_88779_MOESM1_ESM.docx]

# Pesticides in a case study on no-tillage farming systems and surrounding forest patches in Brazil

**Karlo Alves da Silva^1^, Vitoria Beltrame Nicola^2^, Rafaela Tavares Dudas^1^, Wilian Carlo Demetrio^3^, Lilianne dos Santos Maia^3^, Luis Cunha^4,5^, Marie Luise Carolina Bartz^1,4^, George Gardner Brown^3,6^, Amarildo Pasini^7^, Peter Kille^8^, Nuno Gonçalo de Carvalho Ferreira^8*^, Cíntia Mara Ribas de Oliveira^1,2*^**.

^1^ Universidade Positivo, Programa de Pós-Graduação em Gestão Ambiental, Curitiba, 81280-330, Brasil.

^2^ Universidade Positivo, Graduação em Biomedicina, Curitiba, 81280-330, Brasil.

^3^ Universidade Federal do Paraná, Programa de Pós-Graduação em Ciências do Solo, Curitiba, 80035-050, Brasil.

^4^ University of Coimbra, Centre for Functional Ecology, Department of Life Sciences, Calçada Martim de Freitas, 3000-456 Coimbra, Portugal.

^5^ School of Applied Sciences, University of South Wales, Pontypridd, Wales, CF37 4BD UK.

^6^ Embrapa Florestas, Colombo, Paraná, 83411-000, Brasil.

^7^ Universidade Estadual de Londrina, Departamento de Agronomia, Londrina, 86057-970, Brasil.

^8^ Cardiff University, School of Biosciences, Cardiff, CF10 3AX, UK.

*corresponding author [ferreiran@cardiff.ac.uk](mailto:ferreiran@cardiff.ac.uk) and [cmara@up.edu.br](mailto:cmara@up.edu.br)

**SUPPLEMENTARY DATA**

Summary

[**Table S1:** Soil physical and chemical properties of each transect in no-tillage (NT) and secondary forest (SF) sampling sites (A, B and C). N: nitrogen; C: carbon; H: hydrogen; S: sulfur; pH CaCl_2_: hydrogen potential in calcium chloride; Al^3+^: aluminum; H^+^Al: exchange aluminum; Ca^2+^: calcium; Mg^2+^: magnesium; K^+^: potassium; P: phosphorus; CEC: cation exchange capacity. Data presents average values ± standard deviation and [minimum – maximum] values (n = 9). 4](#_Toc66795262)

[**Table S2**: Pesticides in soil samples collected in no-tillage (NT) areas and secondary Atlantic forests (SF) in three sites (A, B and C). Frequency of each active ingredient or metabolite per locality considering (+) as presence and (-) as absence. 5](#_Toc66795263)

[**Table S3:** Spearman correlation between soil properties, glyphosate (GLY) and aminomethylphosphonic acid (AMPA) values. Bold values indicate significant differences at a significance level α = 0.05. 7](#_Toc66795264)

[**Table S4:** Rainfall (mm/d) from January to May 2018. Average daily, maximum rainfall, and rainy days each month for areas A, B, and C. NA: Not evaluated. Data obtained from the Hydrological Information System of the Paraná Water Institute (Secretariat of Environment and Water Resources). 8](#_Toc66795265)

[**Table S5:** Details of analytical methods by high-performance liquid chromatography method. 9](#_Toc66795266)

[**Table S6:** Packages used for statistical computing in R software version 4.0.3 (R Core Team, 2020. R: A Language and Environment for Statistical Computing. R Foundation for Statistical Computing, Vienna, Austria. http://www.R-project.org/). 10](#_Toc66795267)

[**Table S7:** Parameters used to estimate the risk to human health, according to Qu et al. (2019)^11^, ATSDR (2019)^10^ and OEHHA (2019)^11^. 11](#_Toc66795268)

[**Figure S1:** Pearson correlation and coefficient of determination between glyphosate (GLY) and aminomethylphosphonic acid (AMPA) concentrations in soil samples collected in no-tillage areas and in secondary forests. 13](#_Toc66795269)

[**Figure S2:** Σ-modified glycine (C_GLY_ + C_AMPA_) composition in soil samples in no-tillage farms (NT) and secondary Atlantic forest fragments (SF) for each study site (A, B and C). 14](#_Toc66795270)

[**Figure S3:** Field areas sampled in site A (23°56'9.42"S, 51°20'13.50"W). Three transects (9 samples each) in area under no-tillage system (NT) were selected throughout an altitudinal gradient (1 – up land, 2 – mid slope and 3 – low land) and one transect (9 samples) in the surrounding secondary forest (SF). Glyphosate (GLY) and aminomethylphosphonic acid (AMPA) are shown in mg/kg soil. Map data: ©2021Google, Maxar Technologies. 15](#_Toc66795271)

[**Figure S4:** Field areas sampled in site B (23°54'14.11"S, 51°13'24.15"W). Three transects (9 samples each) in area under no-tillage system (NT-B) were selected throughout an altitudinal gradient (1 – up land, 2 – mid slope and 3 – low land) and one transect (9 samples) in the surrounding secondary forest (SF-B). Glyphosate (GLY) and aminomethylphosphonic acid (AMPA) are shown in mg/kg soil. Map data: ©2021Google, Maxar Technologies. 16](#_Toc66795272)

[**Figure S5:** Field areas sampled in site C (25°25'34.87"S, 50°0'12.39"W). Three transects (9 samples each) in area under no-tillage system (NT-C) were selected throughout an altitudinal gradient (1 – up land, 2 – mid slope and 3 – low land) and one transect (9 samples) in the surrounding secondary forest (SF-C). Glyphosate (GLY) and aminomethylphosphonic acid (AMPA) are shown in mg/kg soil. Map data: ©2021Google, Maxar Technologies. 17](#_Toc66795273)

[**References** 18](#_Toc66795274)

# Table S1: Soil physical and chemical properties of each transect in no-tillage (NT) and secondary forest (SF) sampling sites (A, B and C). N: nitrogen; C: carbon; H: hydrogen; S: sulfur; pH CaCl_2_: hydrogen potential in calcium chloride; Al^3+^: aluminum; H^+^Al: exchange aluminum; Ca^2+^: calcium; Mg^2+^: magnesium; K^+^: potassium; P: phosphorus; CEC: cation exchange capacity. Data presents average values ± standard deviation and [minimum – maximum] values (n = 9).

| **Parameter** | **SITE A** | | | | **SITE B** | | | | **SITE C** | | | |
| --- | --- | --- | --- | --- | --- | --- | --- | --- | --- | --- | --- | --- |
|  | **NT-A1** | **NT-A2** | **NT-A3** | **SF-A** | **NT-B1** | **NT-B2** | **NT-B3** | **SF-B** | **NT-C1** | **NT-C2** | **NT-C3** | **SF-C** |
| N (%) | 0.29 ± 0.02  [0.29 – 0.36] | 0.39 ± 0.02  [0.3 – 0.41] | 0.43 ± 0.03  [0.40 – 0.49] | 0.66 ± 0.25  [0.51 – 1.36] | 0.32 ± 0.03  [0.28 – 0.35] | 0.31 ± 0.03  [0.27­ – 0.35] | 0.27 ± 0.02  [0.22 – 0.30] | 0.43 ± 0.05  [0.35 – 0.54] | 0.13 ± 0.04  [0.07 – 0.19] | 0.21 ± 0.03  [0.16 – 0.26] | 0.13 ± 0.02  [0.08 – 0.16] | 0.25 ± 0.02  [0.22 – 0.29] |
| C (%) | 4.50 ± 0.43  [4.05 – 5.21] | 5.34 ± 0.34  [4.83 – 5.92] | 6.46 ± 0.50  [5.86 – 7.33] | 6.27 ± 2.61  [4.8 – 13.52] | 3.79 ± 0.23  [3.45 – 4.09] | 3.39 ± 0.31  [3.00 – 3.75] | 2.93 ± 0.36  [2.24 – 3.48] | 4.80 ± 0.87  [3.55 – 6.65] | 1.17 ± 0.48  [0.48 – 1.95] | 2.15 ± 0.35  [1.65 – 2.66] | 1.24 ± 0.24  [0.86 – 1.61] | 2.46 ± 0.19  [2.09 – 2.84] |
| H (%) | 2.08 ± 0.09  [1.88 – 2.17] | 2.06 ± 0.06  [1.98 – 2.16] | 2.06 ± 0.07  [1.93 – 2.17] | 2.07 ± 0.35  [1.79 – 3.03] | 1.99 ± 0.06  [1.89 – 2.07] | 1.69 ± 0.17  [1.43 – 1.95] | 1.35 ± 0.09  [1.21 – 1.52] | 1.85 ± 0.13  [1.64 – 2.09] | 0.09 ± 0.05  [0.02 – 0.18] | 0.40 ± 0.10  [0.27 – 0.52] | 0.10 ± 0.04  [0.05 – 0.18] | 0.29 ± 0.04  [0.22 – 0.38] |
| S (%) | 0.10 ± 0.02  [0.07 – 0.14] | 0.61 ± 0.78  [0.06 – 2.07] | 0.09 ± 0.02  [0.06 – 0.12] | 0.10 ± 0.01  [0.08 – 0.12] | 0.05 ± 0.00  [0.04 – 0.05] | 0.05 ± 0.01  [0.04 – 0.07] | 0.05 ± 0.01  [0.04 – 0.07] | 0.07 ± 0.03  [0.05 – 0.14] | 0.32 ± 0.74  [0.04 – 2.30] | 0.28 ± 0.56  [0.08 – 1.78] | 0.07 ± 0.01  [0.06 – 0.08] | 0.03 ± 0.00  [0.02 – 0.03] |
| pH CaCl_2_ | 5.27 ± 0.24  [4.83 – 5.59] | 5.31 ± 0.07  [5.22 – 5.44] | 5.42 ± 0.07  [5.30 – 5.54] | 5.20 ± 0.42  [4.43 – 5.87] | 4.87 ± 0.19  [4.61 – 5.20] | 4.90 ± 0.20  [4.63 – 5.20] | 4.82 ± 0.19  [4.53 – 5.11] | 4.12 ± 0.18  [3.90 – 4.59] | 5.38 ± 0.15  [5.17 – 5.63] | 5.73 ± 0.26  [5.13 – 6.02] | 5.56 ± 0.16  [5.33 – 5.87] | 3.85 ± 0.18  [3.46 – 4.15] |
| Al^3+^ (cmolc/dm^3^ soil) | 0.05 ± 0.03  [0.02 – 0.14] | 0.05 ± 0.02  [0.02 – 0.09] | 0.07 ± 0.06  [0.02 – 0.20] | 0.15 ± 0.19  [0.00 – 0.66] | 0.12 ± 0.07  [0.05 – 0.26] | 0.14 ± 0.09  [0.04 – 0.30] | 0.16 ± 0.12  [0.04 – 0.38] | 1.15 ± 0.57  [0.15 – 2.06] | 0.05 ± 0.04  [0.00 – 0.13] | 0.02 ± 0.06  [0.00 – 0.18] | 0.01 ± 0.02  [0.00 – 0.05] | 1.65 ± 0.63  [0.46 – 2.85] |
| H^+^Al (cmolc/dm^3^ soil) | 4.86 ± 0.91  [3.7 – 6.2] | 5.32 ± 0.60  [4.3 – 5.8] | 10.03 ± 12.97  [5.0 – 44.6] | 5.33 ± 1.72  [3.2 – 9.0] | 5.97 ± 0.80  [4.6 – 7.2] | 5.38 ± 0.76  [4.3 – 6.7] | 5.43 ± 0.76  [4.3 – 6.7] | 8.91 ± 1.31  [6.2 – 10.5] | 2.84 ± 1.02  [2.0 – 5.4] | 3.63 ± 0.52  [2.7 – 4.3] | 2.94 ± 0.53  [2.4 – 4.0] | 7.49 ± 1.23  [6.2 – 10.5] |
| Ca^2+^ (cmolc/dm^3^ soil) | 7.61 ± 0.59  [6.83 – 8.98] | 8.02 ± 1.14  [6.15 – 9.83] | 7.54 ± 1.53  [5.00 – 9.75] | 6.76 ± 4.37  [0.95 – 14.03] | 3.67 ± 0.65  [2.58 – 4.78] | 4.72 ± 1.08  [3.30 – 6.68] | 4.42 ± 1.41  [2.80 – 7.28] | 1.96 ± 1.15  [0.43 – 4.7] | 1.92 ± 0.56  [0.78 – 2.73] | 1.63 ± 0.88  [0.25 – 2.65] | 1.46 ± 0.54  [0.50 – 2.33] | 0.50 ± 0.52  [0.08 – 1.83] |
| Mg^2+^ (cmolc/dm^3^ soil) | 2.19 ± 0.27  [1.76 – 2.60] | 2.26 ± 0.26  [1.92 – 2.64] | 2.87 ± 1.11  [0.32 – 3.68] | 3.30 ± 1.20  [1.40 – 5.32] | 1.94 ± 0.29  [1.52 – 2.32] | 1.95 ± 0.34  [1.56 – 2.60] | 1.98 ± 0.48  [1.40 – 2.60] | 1.39 ± 0.46  [0.68 – 2.40] | 2.26 ± 0.26  [1.88 – 2.80] | 1.77 ± 0.59  [0.64 – 2.68] | 1.94 ± 0.37  [1.32 – 2.28] | 0.61 ± 0.33  [0.28 – 1.48] |
| K^+^ (mg/dm^3^ soil) | 0.06 ± 0.02  [0.04 – 0.10] | 0.07 ± 0.02  [0.04 – 0.11] | 0.05 ± 0.02  [0.01 – 0.08] | 0.03 ± 0.01  [0.02 – 0.05] | 0.05 ± 0.01  [0.03 – 0.07] | 0.06 ± 0.02  [0.03 – 0.09] | 0.05 ± 0.01  [0.04 – 0.07] | 0.03 ± 0.01  [0.02 – 0.05] | 0.02 ± 0.01  [0.01 – 0.04] | 0.04 ± 0.01  [0.03 – 0.05] | 0.02 ± 0.01  [0.01 – 0.03] | 0.01 ± 0.00  [0.01 – 0.02] |
| P (mg/dm^3^ soil) | 17.50 ± 8.01  [8.7 – 35.2] | 21.00 ± 3.96  [15.9 – 26.8] | 19.87 ± 7.20  [9.4 – 33.3] | 4.51 ± 1.73  [3.1 – 9.1] | 33.28 ± 7.16  [21.8 – 42.3] | 34.74 ±10.98  [22.7 – 58.3] | 33.27 ± 9.74  [16.4 – 50.9] | 8.26 ± 6.27  [3.5 – 24.7] | 58.4 ± 25.08  [8.2 – 88.9] | 40.36 ± 12.3  [22.7 – 59.4] | 79.44 ± 23.59  [37.8 – 109.4] | 10.91 ± 1.46  [9.10 – 14.2] |
| CEC | 9.91 ± 0.79  [8.93 – 11.67] | 10.40 ± 1.18  [8.87 – 12.42] | 10.53 ± 2.33  [7.42 – 13.56] | 10.23 ± 5.38  [3.03 – 19.16] | 5.79 ± 0.85  [4.40 – 7.22] | 6.87 ± 1.34  [5.11 – 9.44] | 6.62 ± 1.70  [4.62 – 9.62] | 4.53 ± 1.11  [3.18 – 7.30] | 4.25 ± 0.79  [2.72 – 5.54] | 3.46 ± 0.96  [1.96 – 5.02] | 3.44 ± 0.78  [1.95 – 4.54] | 2.77 ± 0.63  [1.82 – 3.78] |
| Clay (g/kg soill) | 587.5 ± 30.6  [537 – 637] | 561.1 ± 52.1  [462 – 625] | 495.8 ± 60.9  [437 – 587] | 713.9 ± 39.3  [662 – 762] | 727.8 ± 28.5  [675 – 762] | 706.4 ± 20.8  [675 – 737] | 591.7 ± 21.7  [575 – 637] | 673.60 ± 10.6  [662 – 687] | 223.6 ± 27.6  [175 – 262] | 113.9 ± 18.2  [87 – 137] | 122.2 ± 10.4  [112 – 137] | 178.6 ± 22.4  [150 – 212] |
| Silt (g/kg soil) | 204.2 ± 21.7  [175 – 250] | 186.1 ± 9.8  [175 – 200] | 197.2 ± 20.5  [175 – 225] | 230.6 ± 31.8  [162 – 275] | 155.6 ± 17.8  [125 – 187] | 162.5 ± 18.8  [137 – 187] | 119.4 ± 25.9  [87 – 175] | 186.1± 17.1  [162 – 212] | 76.4 ± 14.6  [50 – 100] | 25.0 ± 18.8  [12 – 62] | 30.6 ± 16.7  [12 – 50] | 91.1 ± 14.1  [62 – 112] |
| Sand (g/kg soil) | 208.3 ± 17.7  [175 – 225] | 252.8 ± 49.1  [187 – 337] | 306.9 ± 47.6  [237 – 362] | 55.6 ± 40.9  [12 – 150] | 116.7 ± 25.0  [87 – 162] | 130.6 ± 11.0  [112 – 137] | 288.9 ± 30.9  [237 – 337] | 140.3 ± 21.9  [100 – 175] | 700.0 ± 37.5  [650 – 762] | 861.1 ± 24.6  [812 – 887] | 847.22 ± 12.15  [825 – 862] | 730.4 ± 30.0  [687 – 787] |

# Table S2: Pesticides in soil samples collected in no-tillage (NT) areas and secondary Atlantic forests (SF) in three sites (A, B and C). Frequency of each active ingredient or metabolite per locality considering (+) as presence and (-) as absence.

| Chemical group | Active ingredient or metabolite | SF-A | SF-B | SF-C | NT-A | NT-B | NT-C |
| --- | --- | --- | --- | --- | --- | --- | --- |
| Carbamate | m-Cumenyl methyl carbamate | - | - | - | - | - | - |
| Carbamate | Metolcarb | - | - | - | - | - | - |
| Carbamate | Mexacarbate | - | - | - | - | - | - |
| Carbamate | Promecarbe | - | - | - | - | - | - |
| Carbamate | Tiodiocarbe | - | - | - | - | - | - |
| Carbamate | Dioxicarbe | - | - | - | - | - | - |
| Dinitroaniline | Pendimenthalin | - | - | - | - | - | - |
| Organochlorine | Alachlor | - | - | - | - | - | - |
| Organochlorine | Aldrin | - | - | - | - | - | - |
| Organochlorine | Aldrin + Dieldrin | - | - | - | - | - | - |
| Organochlorine | alpha-hexachlorocyclohexane | - | - | - | - | - | - |
| Organochlorine | beta-hexachlorocyclohexane | - | - | - | - | - | - |
| Organochlorine | Cis-Chlordane (alpha) | - | - | - | - | - | - |
| Organochlorine | Chlordane (Cis+Trans) | - | - | - | - | - | - |
| Organochlorine | p,p'-dichlorodiphenyltrichloroethane (DDT) | - | - | - | + | - | - |
| Organochlorine | p,p'-dichlorodiphenyldichloroethylene (DDE) | - | - | - | + | + | - |
| Organochlorine | p,p'-dichlorodiphenyldichloroethane (DDE) | - | - | - | + | + | - |
| Organochlorine | DDTs (DDT+DDE+DDD) | - | - | - | + | + | - |
| Organochlorine | delta-hexachlorocyclohexane | - | - | - | - | - | - |
| Organochlorine | Dieldrin | - | - | - | - | - | - |
| Organochlorine | Endosulfans (I + II + sulfate) | - | - | - | - | - | - |
| Organochlorine | Endosulfan I | - | - | - | - | - | - |
| Organochlorine | Endolsulfan II | - | - | - | - | - | - |
| Organochlorine | Endosulfan sulfate | - | - | - | - | - | - |
| Organochlorine | Endrin | - | - | - | - | - | - |
| Organochlorine | Endrin aldehyde | - | - | - | - | - | - |
| Organochlorine | Endrin ketone | - | - | - | - | - | - |
| Organochlorine | gama-hexachlorocyclohexane | - | - | - | - | - | - |
| Organochlorine | Heptachlor | - | - | - | - | - | - |
| Organochlorine | Heptachlor + Heptachlor epoxide | - | - | - | - | - | - |
| Organochlorine | Heptachlor epoxide | - | - | - | - | - | - |
| Organochlorine | Hexachlorobenzene | - | - | - | - | - | - |
| Organochlorine | Metolachlor | - | - | - | - | - | - |
| Organochlorine | Metoxichlor | - | - | - | - | - | - |
| Organochlorine | Toxaphene | - | - | - | - | - | - |
| Organochlorine | Trans-chlordane (gama) | - | - | - | - | - | - |
| Organochlorine | Trifluraline | - | - | - | - | - | - |
| Organophosphate | Dementon-s | - | - | - | - | - | - |
| Organophosphate | Parathion | - | - | - | - | - | - |
| Organophosphate | Ethyl parathion | - | - | - | - | - | - |
| Organophosphate | Methyl parathion | - | - | - | - | - | - |
| Organophosphate | Chlorpyrifos | - | - | - | - | - | - |
| Chemical group | **Active ingredient or metabolite** | **SF-A** | **SF-B** | **SF-C** | **NT-A** | **NT-B** | **NT-C** |
| Organophosphate | Chlorpyrifos + chlorpyrifos-oxon | - | - | - | - | - | - |
| Organophosphate | Chlorpyrifos-oxon | - | - | - | - | - | - |
| Organophosphate | Dementon-o | - | - | - | - | - | - |
| Organophosphate | Dementon (Dementon-o + Dementon-s) | - | - | - | - | - | - |
| Organophosphate | Azinphos methyl | - | - | - | - | - | - |
| Organophosphate | Malathion | - | - | - | - | - | - |
| Organophosphate | Profenofos | - | - | - | - | - | - |
| Organophosphate | Terbufos | - | - | - | - | - | - |
| Pyrethroid | Permethrin | - | - | - | - | - | - |
| Triazine | Atrazine | - | - | - | - | - | - |
| Triazine | Simazine | - | - | - | - | - | - |

# Table S3: Spearman correlation between soil properties, glyphosate (GLY) and aminomethylphosphonic acid (AMPA) values. Bold values indicate significant differences at a significance level α = 0.05.

| **Group** | **Parameter** | **N** | **C** | **H** | **S** | **pH** | **Al^3+^** | **H^+^Al** | **Ca^2+^** | **Mg^2+^** | **K^+^** | **P** | **Clay** | **Silt** | **Sand** | **CEC** | **GLY** | **AMPA** | **AMPA:GLY ratio** | **TEG** | **% AMPA** | **% GLY** |
| --- | --- | --- | --- | --- | --- | --- | --- | --- | --- | --- | --- | --- | --- | --- | --- | --- | --- | --- | --- | --- | --- | --- |
| **PHYSICAL-CHEMICAL PROPERTIES** | **N** |  |  |  |  |  |  |  |  |  |  |  |  |  |  |  |  |  |  |  |  |  |
|  | **C** | **0.96** |  |  |  |  |  |  |  |  |  |  |  |  |  |  |  |  |  |  |  |  |
|  | **H** | **0.83** | **0.91** |  |  |  |  |  |  |  |  |  |  |  |  |  |  |  |  |  |  |  |
|  | **S** | **0.29** | **0.35** | **0.33** |  |  |  |  |  |  |  |  |  |  |  |  |  |  |  |  |  |  |
|  | **pH** | **-0.20** | -0.09 | -0.04 | **0.57** |  |  |  |  |  |  |  |  |  |  |  |  |  |  |  |  |  |
|  | **Al^3+^** | **0.27** | 0.17 | 0.09 | **-0.49** | **-0.88** |  |  |  |  |  |  |  |  |  |  |  |  |  |  |  |  |
|  | **H^+^Al** | **0.51** | **0.46** | **0.35** | **-0.30** | **-0.79** | **0.78** |  |  |  |  |  |  |  |  |  |  |  |  |  |  |  |
|  | **Ca^2+^** | **0.60** | **0.72** | **0.77** | **0.42** | **0.27** | **-0.26** | -0.06 |  |  |  |  |  |  |  |  |  |  |  |  |  |  |
|  | **Mg^2+^** | **0.29** | **0.35** | **0.35** | **0.39** | **0.52** | **-0.51** | **-0.44** | **0.65** |  |  |  |  |  |  |  |  |  |  |  |  |  |
|  | **K^+^** | **0.20** | **0.33** | **0.47** | 0.17 | 0.13 | **-0.23** | -0.08 | **0.63** | **0.31** |  |  |  |  |  |  |  |  |  |  |  |  |
|  | **P** | **-0.66** | **-0.60** | **-0.46** | -0.07 | **0.37** | **-0.47** | **-0.55** | -0.17 | 0.03 | 0.16 |  |  |  |  |  |  |  |  |  |  |  |
|  | **Clay** | **0.63** | **0.54** | **0.56** | -0.15 | **-0.47** | **0.42** | **0.46** | **0.38** | 0.08 | **0.29** | **-0.38** |  |  |  |  |  |  |  |  |  |  |
|  | **Silt** | **0.86** | **0.85** | **0.80** | **0.28** | -0.18 | **0.21** | **0.40** | **0.67** | **0.37** | **0.28** | **-0.61** | **0.63** |  |  |  |  |  |  |  |  |  |
|  | **Sand** | **-0.71** | **-0.61** | **-0.61** | 0.05 | **0.43** | **-0.39** | **-0.45** | **-0.42** | -0.14 | **-0.25** | **0.47** | **-0.97** | **-0.75** |  |  |  |  |  |  |  |  |
|  | **CEC** | **0.65** | **0.75** | **0.78** | **0.38** | **0.22** | **-0.20** | -0.02 | **0.98** | **0.70** | **0.59** | **-0.24** | **0.42** | **0.72** | **-0.47** |  |  |  |  |  |  |  |
| **PESTICIDES VALUES** | **GLY** | 0.13 | 0.06 | 0.02 | **-0.26** | **-0.32** | **0.35** | 0.17 | -0.06 | -0.12 | -0.07 | -0.12 | **0.31** | 0.08 | **-0.29** | -0.03 |  |  |  |  |  |  |
|  | **AMPA** | 0.12 | 0.12 | 0.16 | -0.12 | -0.07 | 0.08 | 0.00 | **0.23** | 0.11 | 0.17 | 0.12 | **0.37** | 0.18 | **-0.32** | **0.23** | **0.58** |  |  |  |  |  |
|  | **AMPA:GLY ratio** | 0.05 | 0.11 | 0.14 | 0.05 | 0.14 | -0.17 | -0.07 | **0.23** | **0.19** | 0.18 | 0.10 | 0.09 | 0.14 | -0.08 | **0.24** | **-0.43** | **0.33** |  |  |  |  |
|  | **TEG** | 0.14 | 0.07 | 0.05 | **-0.24** | **-0.26** | **0.29** | 0.12 | 0.03 | -0.04 | 0.00 | -0.04 | **0.36** | 0.11 | **-0.33** | 0.05 | **0.94** | **0.80** | -0.19 |  |  |  |
|  | **% AMPA** | 0.05 | 0.11 | 0.14 | 0.05 | 0.14 | -0.17 | -0.07 | **0.23** | **0.19** | 0.18 | 0.10 | 0.09 | 0.14 | -0.08 | **0.24** | **-0.43** | **0.33** | **1.00** | -0.19 |  |  |
|  | **% GLY** | -0.05 | -0.11 | -0.14 | -0.05 | -0.14 | 0.17 | 0.07 | **-0.23** | **-0.19** | -0.18 | -0.10 | -0.09 | -0.14 | 0.08 | **-0.24** | **0.43** | **-0.33** | **-1.00** | 0.19 | **-1.00** |  |

# Table S4: Rainfall (mm/d) from January to May 2018. Average daily, maximum rainfall, and rainy days each month for areas A, B, and C. NA: Not evaluated. Data obtained from the Hydrological Information System of the Paraná Water Institute (Secretariat of Environment and Water Resources).

| **Site** | **Month** | **Total rainfall (mm)** | **Average rainfall (mm/d)** | **Maximum rainfall (mm)** | **Rainy d** |
| --- | --- | --- | --- | --- | --- |
| **NT-A**  **&**  **SF-A** | Jan | 374.20 | 12.07 | 43.7 | 25 |
|  | Feb | 100.70 | 3.60 | 35.2 | 9 |
|  | Mar | 246.10 | 7.94 | 67 | 15 |
|  | Apr | 13.20 | 0.44 | 8.7 | 5 |
|  | May | 38.00 | 1.23 | 26.6 | 5 |
| **NT-B**  **&**  **SF-B** | Jan | 280.80 | 9.06 | 29.5 | 19 |
|  | Feb | 59.40 | 2.12 | 17.1 | 8 |
|  | Mar | 150.20 | 4.85 | 29.5 | 14 |
|  | Apr | 0.00 | 0.00 | 0 | 0 |
|  | May | 11.10 | 0.36 | 11.1 | 1 |
| **NT-C**  **&**  **SF-C** | Jan | 267.50 | 8.63 | 64.9 | 12 |
|  | Feb | 34.80 | 1.24 | 19.8 | 4 |
|  | Mar | NA | NA | NA | NA |
|  | Apr | 13.10 | 0.44 | 10.0 | 2 |
|  | May | 45.00 | 1.45 | 40.0 | 3 |

# Table S5: Details of analytical methods by high-performance liquid chromatography method.

| **Step** | **Procedures details** |
| --- | --- |
| 1 | 10 g of soil samples were extracted with trisodium phosphate (0.03 mol/L Na_3_PO_4_) and sodium citrate (0.01 mol/L Na_3_C_6_H_5_O_7_) solutions in an ultrasonic bath for 30 min. |
| 2 | The solution was centrifuged (3,493 g, 10 min), and the pH of the supernatant was adjusted to 9.0, followed by filtration (0.2 μm quantitative filter). |
| 3 | A liquid-liquid extraction was carried out with the filtrates and n-hexane, and the aqueous phases (1 mL) derivatised using 9-Fluorenylmethyl chloroformate (FMOC-Cl, 1 g/L C_15_H_11_ClO_2_), for 4 h. Samples were then washed with diethyl ether to remove residual FMOC-Cl. |
| 4 | The aqueous phase was filtered on Chromafil Xtra PET membrane (25 mm x 0.2 μm) and analysed for GLY and AMPA contents using HPLC (HPLC LC 20AT, Shimadzu), coupled to fluorescence detector (RF-10A XL, Shimadzu, excitation λ = 254 nm; emission λ = 301 nm). |
| 5 | The mobile phase was programmed over time using as solvent acetonitrile (Phase A, C_2_H_3_N) and 0.2% v/v aqueous phosphoric acid solution (Phase B, H_3_PO_4_). The percentage of Phase B was modified as follows: 0 min - 35%; 10 min - 25%; 15 min - 80%; 20 min - 35%. The flow rate was 1.0 mL/min, with a temperature of 35°C, on a Kromasil C_18_ (5 μm, 250 mm x 4.6 mm) column. |

# Table S6: Packages used for statistical computing in R software version 4.0.3 (R Core Team, 2020. R: A Language and Environment for Statistical Computing. R Foundation for Statistical Computing, Vienna, Austria. http://www.R-project.org/).

| **Package** | **Authors** |
| --- | --- |
| corplot | Wei & Simko (2017)^1^ |
| psych | Revelle (2018)^2^ |
| FactoMiner | Le et al. (2008)^3^ |
| Factoextra | Kassambra & Mundt (2017)^4^ |
| dunn.test | Dinno (2017)^5^ |
| CCA | González & Déjean (2012)^6^ |
| candisc | Friendly & Fox (2017)^7^ |
| yacca | Butts (2017)^8^ |
| ggmap | Kahle & Wickham (2013)^9^ |
| ggplot2 | Wickham (2016)^10^ |

# Table S7: Parameters used to estimate the risk to human health, according to Qu et al. (2019)^11^, ATSDR (2019)^10^ and OEHHA (2019)^11^.

| **Parameter** | **Acronym** | **Unity** | **Value** |
| --- | --- | --- | --- |
| Soil ingestion rate | IRsoil | mg/d | 29.95 |
| Exposure duration | ED | yr | 0,52 |
| Exposure frequency | EF | d/yr | 252 |
| Conversion factor | CF | kg/mg | 1 x 10^-5^ |
| Body weight | BW | kg | 59.78 |
| Average life span | AT | d | 25,55 |
| Bioconcentration factor | BCF | unitless | 0.52 ^a^ |
| Vegetable ingestion rate | IRveget | kg/d | 0.137 |
| Air inhalation rate | IRair | m^3^/d | 32.73 |
| Particle emission factor | PEF | m^3^/kg | 1.36 x 10^9^ |
| Surface area of the skin that contacts soil | SA | cm^2^/d | 18182 |
| Dermal surface factor | AF | mg/cm^2^ | 0.02 |
| Dermal absorption factor | ABS | unitless | 0.13 |
| Oral slope factor | SFo | mg/kg/d | 6.20^-4 b^ |
| Fraction of contaminant absorbed in gastrointestinal tract | ABSgi | unitless | 1 |
| Dermal slope factor | SFabs | mg/kg/d | SFo x ABSgi |
| Inhalation unit risk | IUR | mg/m^3^ | 0 |

^a^ Agency for Toxic Substances and Disease Registry (Registry – U.S. Department of Health and Human Services (ATSDR)^12^.

^b^ California Office of Environmental Health Hazard Assessment (OEHHA)^13^.

The indirect assessment was calculated based on the average daily dose (ADD, mg/kg/d), calculated by four exposure routes: equation (1) for soil ingestion (ing_soil), equation (2) for food ingestion (ing_food), equation (3) for dermal contact (derm) and equation (4) for dust inhalation (inh), expressed as follows:

(1) *ADDing_soil =* $(Csoil x IRsoil x ED x EF x CF)/(BW x AT)$

(2) *ADDing_food =* $(CsoIl x FBC x IRveget x ED x EF)/(BW x AT)$

(3) *ADDderm =* $(Csoil x SA x AF x ABS x ED x EF x CF)/(BW x AT)$

(4) *ADDinh =* $(Csoil x IRar x ED x EF)/(PEF x BW x AT)$

where Csoil is the GLY concentration in soil (mg/kg soil), IRsoil represents the soil ingest rate (mg/d), ED is the exposure duration (yr), EF is the exposure frequency (d/yr), CF is conversion factor (kg/mg), BW is the body weight of the individual adult (kg), AT is the average life (d), BCF is the bioconcentration factor (without unit of measure), IRveget is the daily vegetable ingestion rate (kg/d), SA is the surface area of the skin that contact with the soil (cm^2^/d), AF is the soil skin adhesion factor (mg/cm^2^), ABS is the dermal absorption factor (without unit of measure), IRair represents the air inhalation rate (m^3^/d) and PEF is the particle emission factor (m^3^/kg).

After ADD determination, a cancer slope factor was added to the equations to define the upper confidence limit on the increased cancer risk from each exposure route. The incremental lifetime cancer risk (ILCR) was calculated using the estimated ADD multiplied by the cancer slope factor (*SF*, mg/kg/d) according to equation (5):

(5) *ILCR = ADD* x *SF*

where SF includes oral slope factor (SFo, mg/kg/d), dermal slope factor of dermal contact (SFabs, , mg/kg/d) and the inhalation unit risk (IUR, mg/m^3^). Total lifetime risk of cancer (ILCRs) was calculated by summing the individual ILCR associated with each exposure route (i) according to equation (6):

(6) *ILCRs = ΣILCRi = ΣADDi x SF*


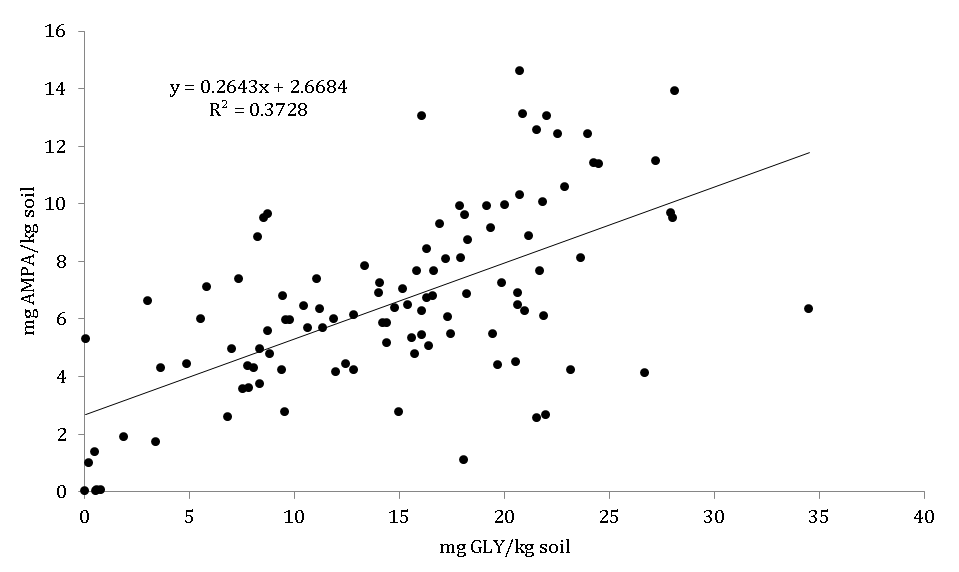


# Figure S1: Pearson correlation and coefficient of determination between glyphosate (GLY) and aminomethylphosphonic acid (AMPA) concentrations in soil samples collected in no-tillage areas and in secondary forests.


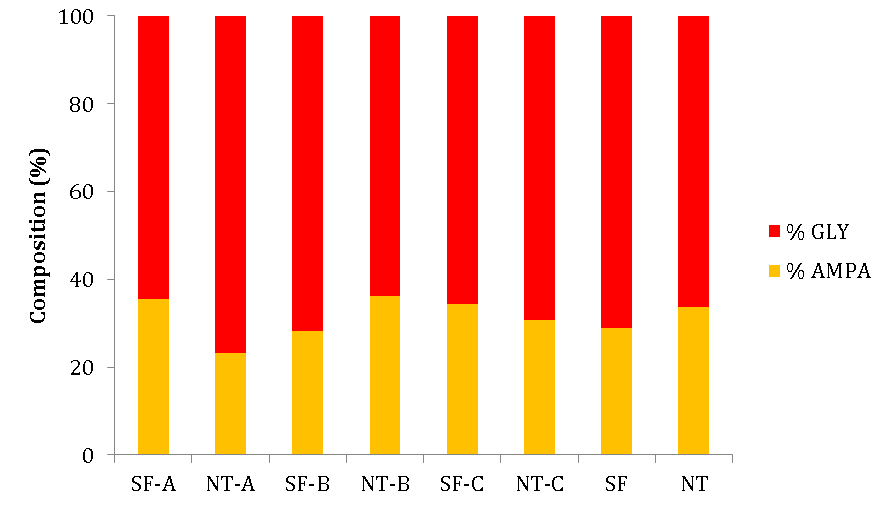


# Figure S2: Σ-modified glycine (C_GLY_ + C_AMPA_) composition in soil samples in no-tillage farms (NT) and secondary Atlantic forest fragments (SF) for each study site (A, B and C).


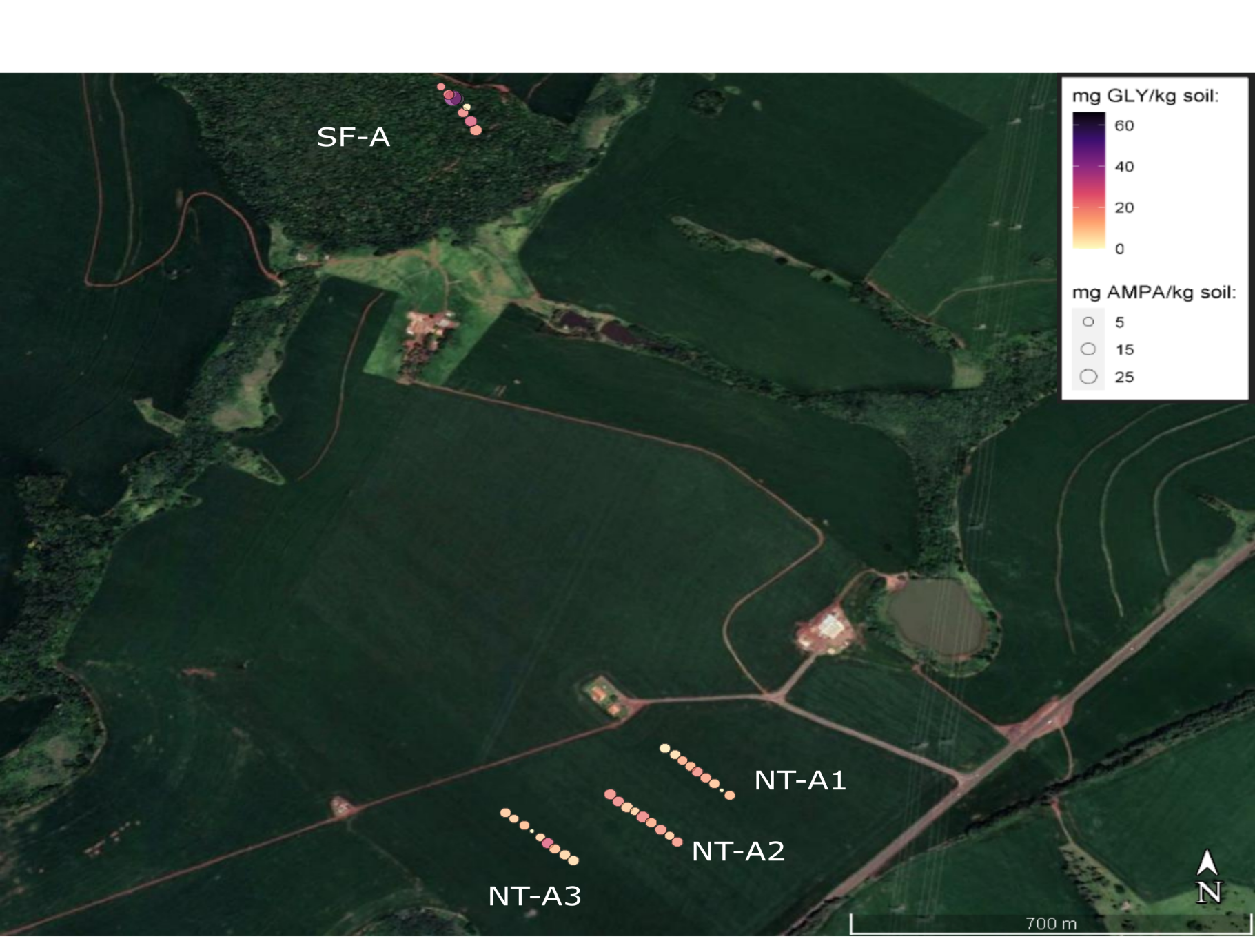


# Figure S3: Field areas sampled in site A (23°56'9.42"S, 51°20'13.50"W). Three transects (9 samples each) in area under no-tillage system (NT) were selected throughout an altitudinal gradient (1 – up land, 2 – mid slope and 3 – low land) and one transect (9 samples) in the surrounding secondary forest (SF). Glyphosate (GLY) and aminomethylphosphonic acid (AMPA) are shown in mg/kg soil. Map data: ©2021Google, Maxar Technologies.


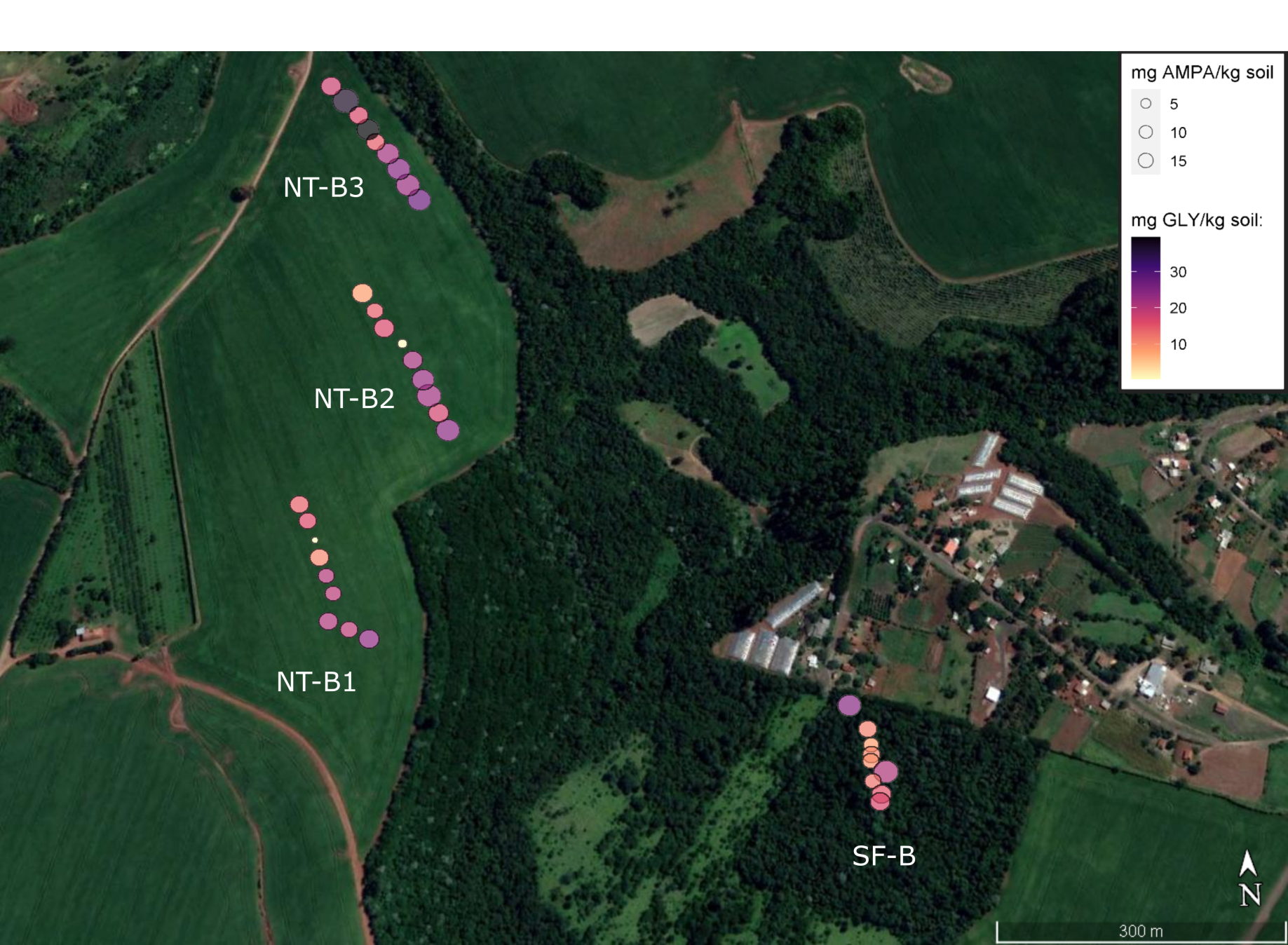


# Figure S4: Field areas sampled in site B (23°54'14.11"S, 51°13'24.15"W). Three transects (9 samples each) in area under no-tillage system (NT-B) were selected throughout an altitudinal gradient (1 – up land, 2 – mid slope and 3 – low land) and one transect (9 samples) in the surrounding secondary forest (SF-B). Glyphosate (GLY) and aminomethylphosphonic acid (AMPA) are shown in mg/kg soil. Map data: ©2021Google, Maxar Technologies.

**
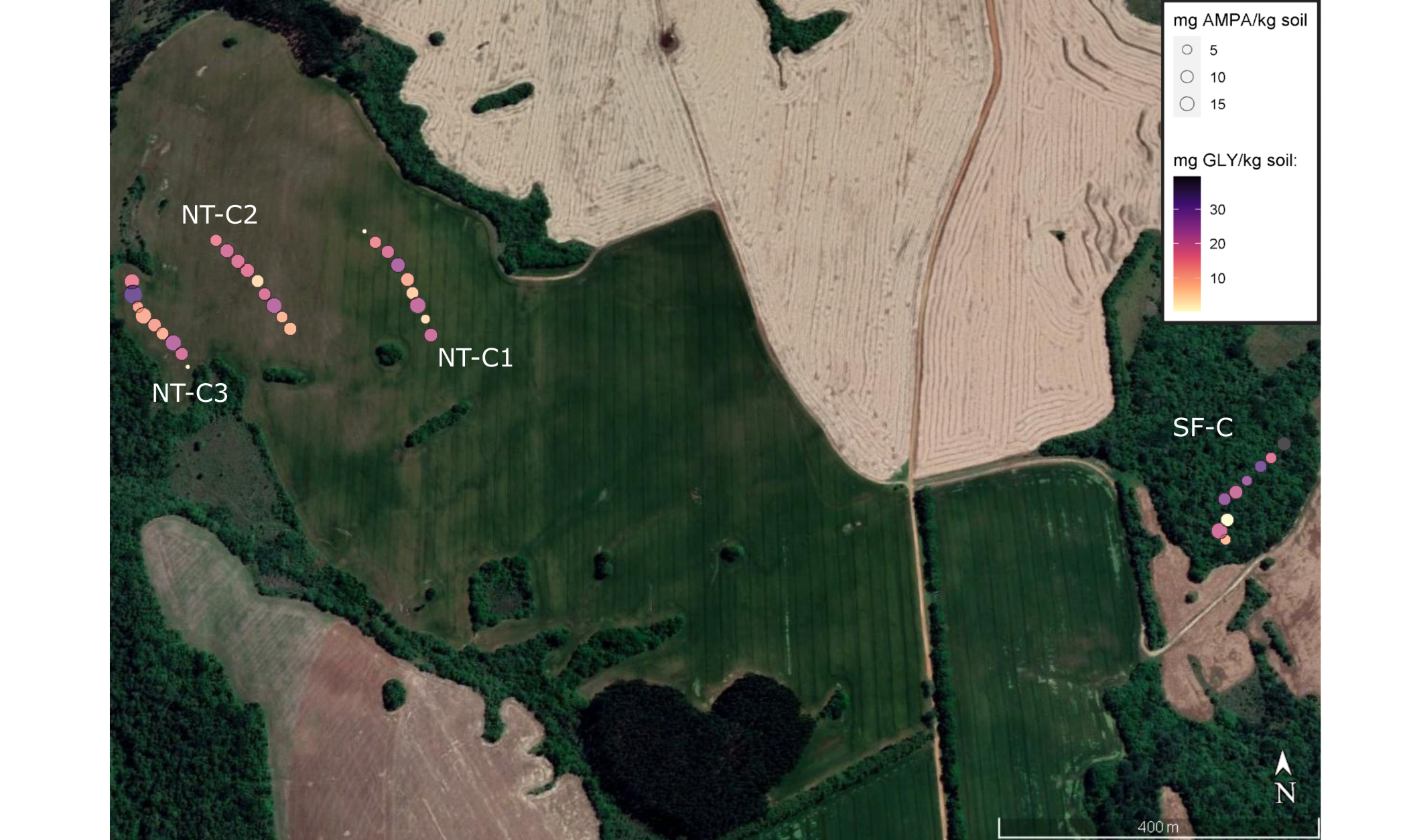
**

# Figure S5: Field areas sampled in site C (25°25'34.87"S, 50°0'12.39"W). Three transects (9 samples each) in area under no-tillage system (NT-C) were selected throughout an altitudinal gradient (1 – up land, 2 – mid slope and 3 – low land) and one transect (9 samples) in the surrounding secondary forest (SF-C). Glyphosate (GLY) and aminomethylphosphonic acid (AMPA) are shown in mg/kg soil. Map data: ©2021Google, Maxar Technologies.

# References

1. Wei, T. & Simko, V. R package ‘corrplot’: Visualization of a Correlation Matrix. (2017). Available at: https://github.com/taiyun/corrplot.

2. Revelle, W. psych: Procedures for Psychological, Psychometric, and Personality Research. (2018). Available at: https://cran.r-project.org/package=psych.

3. Le, S., Josse, J. & Husson, F. FactoMineR: An R Package for Multivariate Analysis. *J. Stat. Softw.* **25**, 1–18 (2008).

4. Kassambara, A. & Mundt, F. factoextra: Extract and Visualize the Results of Multivariate Data Analyses. (2017). Available at: https://cran.r-project.org/package=factoextra.

5. Dinno, A. dunn.test: Dunn’s Test of Multiple Comparisons Using Rank Sums. (2017). Available at: https://cran.r-project.org/package=dunn.test.

6. González, I. & Déjean, S. CCA: Canonical correlation analysis. (2012). Available at: https://cran.r-project.org/package=CCA.

7. Friendly, M. & Fox, J. candisc: Visualizing Generalized Canonical Discriminant and Canonical Correlation Analysis. (2017).

8. Butts, C. T. yacca: Yet Another Canonical Correlation Analysis Package. (2018). Available at: https://cran.r-project.org/package=yacca.

9. Kahle, D. & Wickham, H. ggmap: Spatial Visualization with ggplot2. *R Journal2* **5**, 144–161 (2013).

10. Wickham, H. *ggplot2: Elegant Graphics for Data Analysis*. *Springer-Verlag New York* (2016).

11. Qu, C. *et al.* Organochlorine pesticides in the soils from Benevento provincial territory, southern Italy: Spatial distribution, air-soil exchange, and implications for environmental health. *Sci. Total Environ.* **674**, 159–170 (2019).

12. ATSDR. Agency for Toxicity Substances and Disease Registry. (2019). Available at: https://www.atsdr.cdc.gov/toxprofiles/tp214-c5.pdf. (Accessed: 15th October 2019)

13. OEHHA, C. O. of E. H. H. A. Glyphosate to be Listed under Proposition 65 as Known to the State to Cause Cancer. *2017* (2017). Available at: https://oehha.ca.gov/proposition-65/crnr/glyphosate-be-listed-under-proposition-65-known-state-cause-cancer.
